# Point mutations in *Candida glabrata* 3-hydroxy-3-methylglutaryl-coenzyme A reductase (*Cg*HMGR) decrease enzymatic activity and substrate/inhibitor affinity

**DOI:** 10.1038/s41598-021-00356-w

**Published:** 2021-10-21

**Authors:** Dulce Andrade-Pavón, Vanessa Fernández-Muñoz, Wendy González-Ibarra, César Hernández-Rodríguez, J. Antonio Ibarra, Lourdes Villa-Tanaca

**Affiliations:** 1 Laboratorio de Biología Molecular de Bacterias y Levaduras, Departamento de Microbiología, Escuela Nacional de Ciencias Biológicas, Instituto Politécnico Nacional, CDMX, Prol. de Carpio y Plan de Ayala. Col. Sto. Tomás, CP 11340 Mexico City, Mexico; 2 Laboratorio de Genética Microbiana, Departamento de Microbiología, Escuela Nacional de Ciencias Biológicas, Instituto Politécnico Nacional, CDMX, Mexico City, Mexico

**Keywords:** Microbiology, Molecular biology

## Abstract

3-Hydroxy-3-methylglutaryl-coenzyme A reductase (HMGR) is a crucial enzyme in the ergosterol biosynthesis pathway. The aim of this study was to obtain, purify, characterize, and overexpress five point mutations in highly conserved regions of the catalytic domain of *Candida glabrata* HMGR (*Cg*HMGR) to explore the function of key amino acid residues in enzymatic activity. Glutamic acid (Glu) was substituted by glutamine in the E680Q mutant (at the dimerization site), Glu by glutamine in E711Q (at the substrate binding site), aspartic acid by alanine in D805A, and methionine by arginine in M807R (the latter two at the cofactor binding site). A double mutation, E680Q-M807R, was included. Regarding recombinant and wild-type *Cg*HMGR, in vitro enzymatic activity was significantly lower for the former, as was the in silico binding energy of simvastatin, alpha-asarone and the HMG-CoA substrate. E711Q displayed the lowest enzymatic activity and binding energy, suggesting the importance of Glu^711^ (in the substrate binding site). The double mutant *Cg*HMGR E680Q-M807R exhibited the second lowest enzymatic activity. Based on the values of the kinetic parameters *K*_*M*_ and *V*_*max*_, the mutated amino acids appear to participate in binding. The current findings provide insights into the role of residues in the catalytic site of *Cg*HMGR.

## Introduction

The ergosterol biosynthesis pathway has been proposed as a target for identifying and designing new antifungals. In a rate-limiting step of the biosynthetic pathway of ergosterol in fungi and cholesterol in humans, the enzyme 3-hydroxy-3-methylglutaryl-coenzyme A reductase (HMGR) catalyzes the conversion of 3-hydroxy-3-methylglutaryl-coenzyme A (HMG-CoA) to mevalonate^1^. This pathway also generates other bioactive molecules, including a coenzyme (Q10) that participates in the transport of electrons in mitochondria^2^.

$$ {\text{(S)-HMG-CoA}}\, + \,{\text{2NADPH}}\, + \,{\text{2H}}^{ + } \to {\text{(R)-mevalonate}}\, + \,{\text{2NADP}}^{ + } \, + \,{\text{CoASH}} $$

Phylogenetic analyses have revealed two types of HMGR: class I of eukaryotic organisms and some archaea and class II of prokaryotes and some archaea. Whereas the first class has three domains (catalytic, linker, and membrane-anchor), most members of the second one contain only the catalytic domain^1^.

The HMGR enzyme is the target of lipid-lowering drugs such as statins^3^. These drugs have been described as competitive inhibitors because they dispute the active site of HMGR with the substrate HMG-CoA^1^. Since statins can induce myopathy and hepatotoxicity^4^, new bioactive principles derived from natural sources (e.g., alpha-asarone) have been synthesized. In a murine model, alpha-asarone decreased cholesterol and triglyceride levels. Moreover, its inhibitory effect was tested on the recombinant human HMGR (*h*HMGR) enzyme and an enriched enzyme extract from *Schizosaccharomyces pombe*^5,6^.

The catalytic domain of HMGR of eukaryotic organisms is highly conserved and harbors the dimerization site, the substrate (HMG-CoA) binding site, and the cofactor (NADPH) binding site^1^. Hence, our group has proposed fungal HMGR enzymes as a model for evaluating compounds with antifungal and hypocholesterolemic activity^7^. One of the fungi used for this purpose has been *Candida glabrata* because of its high incidence as an opportunistic pathogenic yeast in hospitalized patients with compromised immunity and its resistance to multiple conventional drugs, including azoles and echinocandins. Such multi-drug resistance has led to treatment failures^8,9^.

In previous research, the catalytic domain of the HMGR enzyme from *C. glabrata* (*Cg*HMGR) was cloned, expressed and characterized, demonstrating its enzymatic activity as well as its inhibition by simvastatin and compounds derived from alpha asarone. Consequently, it has been proposed as a possible antifungal target^10,11^.

The recombinant *Cg*HMGR enzyme is available and its catalytic activity is still under study. Therefore, the aim of the current contribution was to generate, characterize, and overexpress five point mutants of *Cg*HMGR in order to explore the role of certain amino acid residues in the functional capacity of the catalytic domain of *Cg*HMGR. Three highly conserved regions within the catalytic domain were mutated: the dimerization site, the substrate binding site, and the cofactor binding site^12^. The investigation of point mutations in HMGR has been limited, to the best of our knowledge, to humans, *Pseudomonas mevalonii* and Syrian hamsters. They have not been performed on the HMGR of fungi, much less in an opportunistic pathogenic yeast^12–16^.

## Results

### Conservation of the sites of interest in the catalytic domain of HMGRs from different organisms

**Figure 1:**
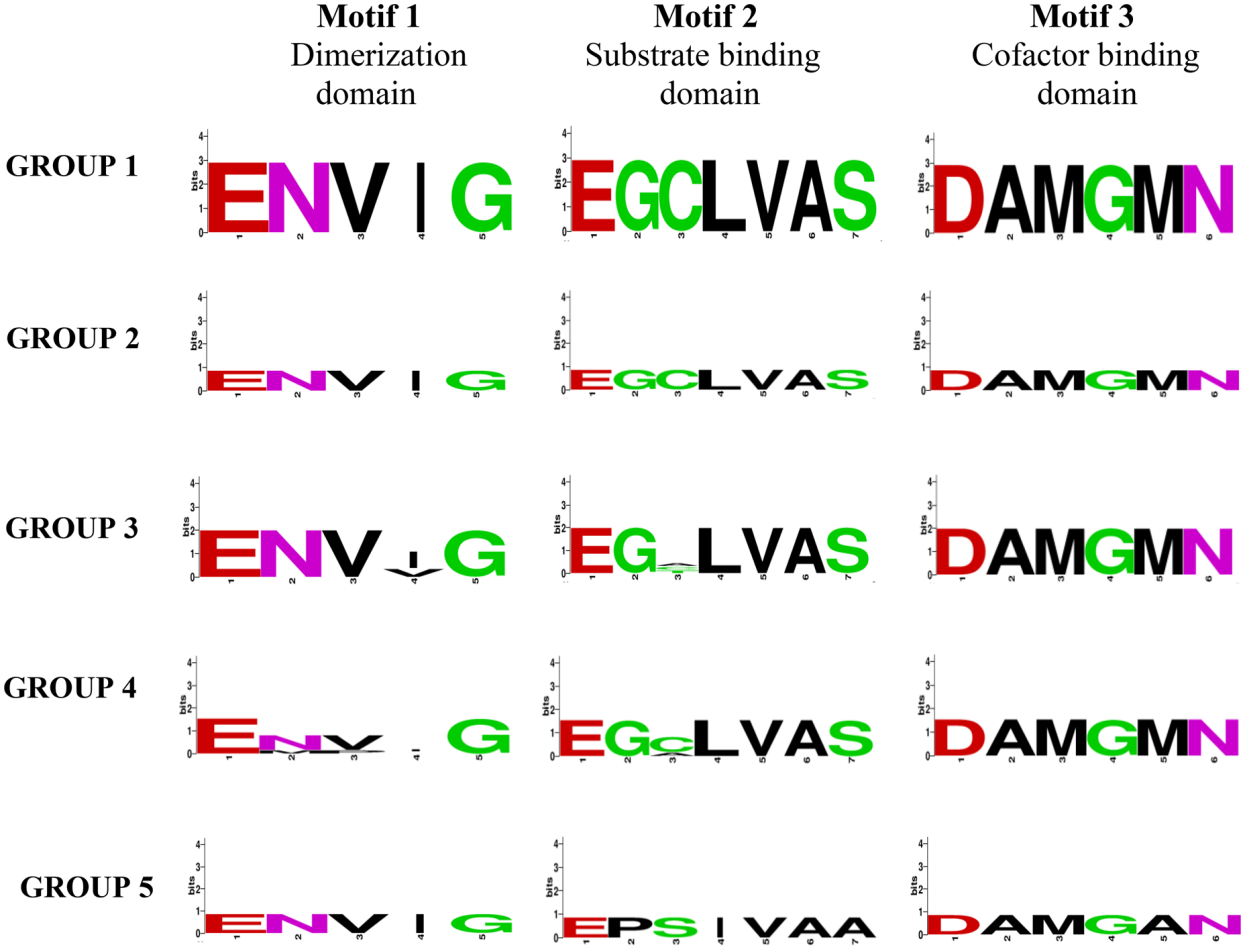
Sequence logos of the conserved motifs found in the catalytic domain of the HMGRs of different organisms. Multiple alignments were performed with Clustal Omega and the consensus logos were generated with WebLogo. Group 1 (*Candida auris*, *Candida haemulonii*, *Clavispora lusitaniae* ATCCC 42720, *Debaryomyces hansenii* CBS767, *Meyerozyma guilliermondii* ATCC 6260, *Candida parapsilosis*, *Candida orthopsilosis* Co 90-125, *Candida tropicalis* MYA-3404, *Candida albicans* SC5314, and *Candida dubliniensis* CD36); group 2 (*C. glabrata* CBS138, *Candida kefyr*, *Kluyveromyces lactis* NRRL Y-1140, *Saccharomyces cerevisiae* S288c HMG1, and *S. cerevisiae* S288c HMG2); group 3 (*Yarrowia lipolytica* CLIB122, *S. pombe*, and *Ustilago maydis*); group 4 (*Drosophila melanogaster*, *Mus musculus*, *H. sapiens*, and *Rattus norvegicus*); and group 5 (*P. mevalonii*).

A multiple alignment was carried out on the Clustal Omega program for the amino acid sequence of the soluble fraction of HMGR from plants, bacteria, insects, mammals (including *Homo sapiens*), and opportunistic pathogenic yeasts (e.g., *C. glabrata*) (Supplementary Fig. S1). The access codes of the corresponding sequences can be consulted in the Materials and Methods section. The motif sequences in the sites of interest in the HMGR enzymes are highlighted in Fig. 1: the dimerization site (ENVIG), the substrate binding site (EGCLVAS), and the cofactor binding site (DAMGMN). The amino acid sequences encoding the soluble fraction of the HMGR enzymes of different organisms are highly conserved, which emphasizes the importance of examining some of the amino acid residues in these sites in order to evaluate their role in the catalysis of the enzyme. Such an analysis has been performed in studies on *P. mevalonii.*

**Figure 2:**
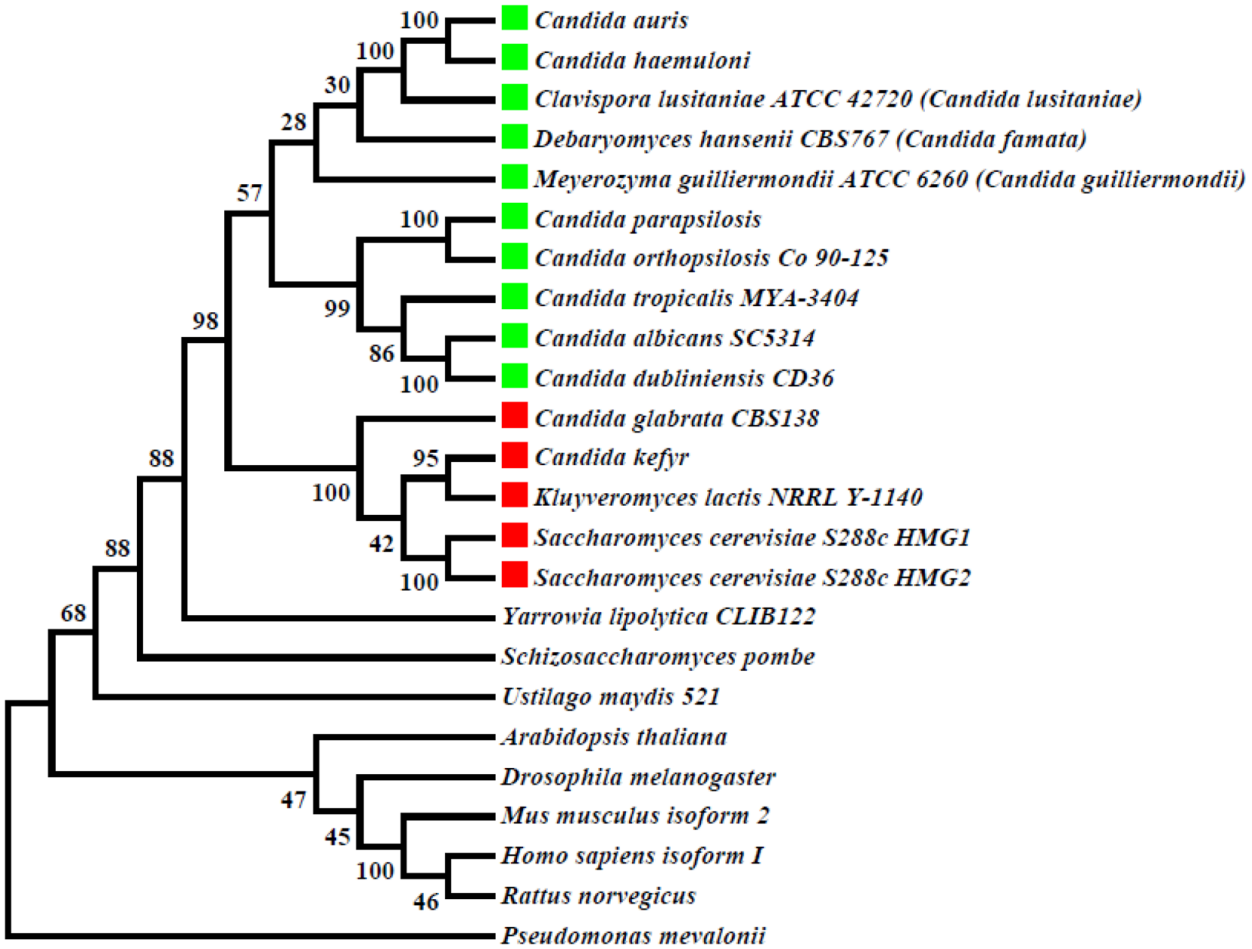
The phylogenetic tree of the catalytic domain of HMGR enzymes in different organisms. The percentage of replicated trees in which the associated taxa are grouped in the 100 bootstrap replicates is shown next to the branches. The phylogenetic tree is drawn to scale, with the length of the branches representing the corresponding evolutionary distances according to the MEGA6 program, the maximum likelihood method, and the WAG + G model. The development of the species in the clade that encompasses *Candida glabrata* (in red) was based on whole genome duplication (WGD). In the clade containing *Candida albicans* (in green), the species developed based on genetic code transition (GCT).

A phylogenetic tree was constructed by examining the amino acid sequences detected in the catalytic domain of the twenty-four organisms included in the alignment test (Fig. 2). The phylogenetic analysis of *Cg*HMGR demonstrated that it belongs to the clade of the yeast species of the Saccharomycotina subphylum and the *Saccharomyces* genus, not to the clade containing *C. albicans.*

### Molecular modeling of wild-type and mutant CgHMGR proteins

**Figure 3:**
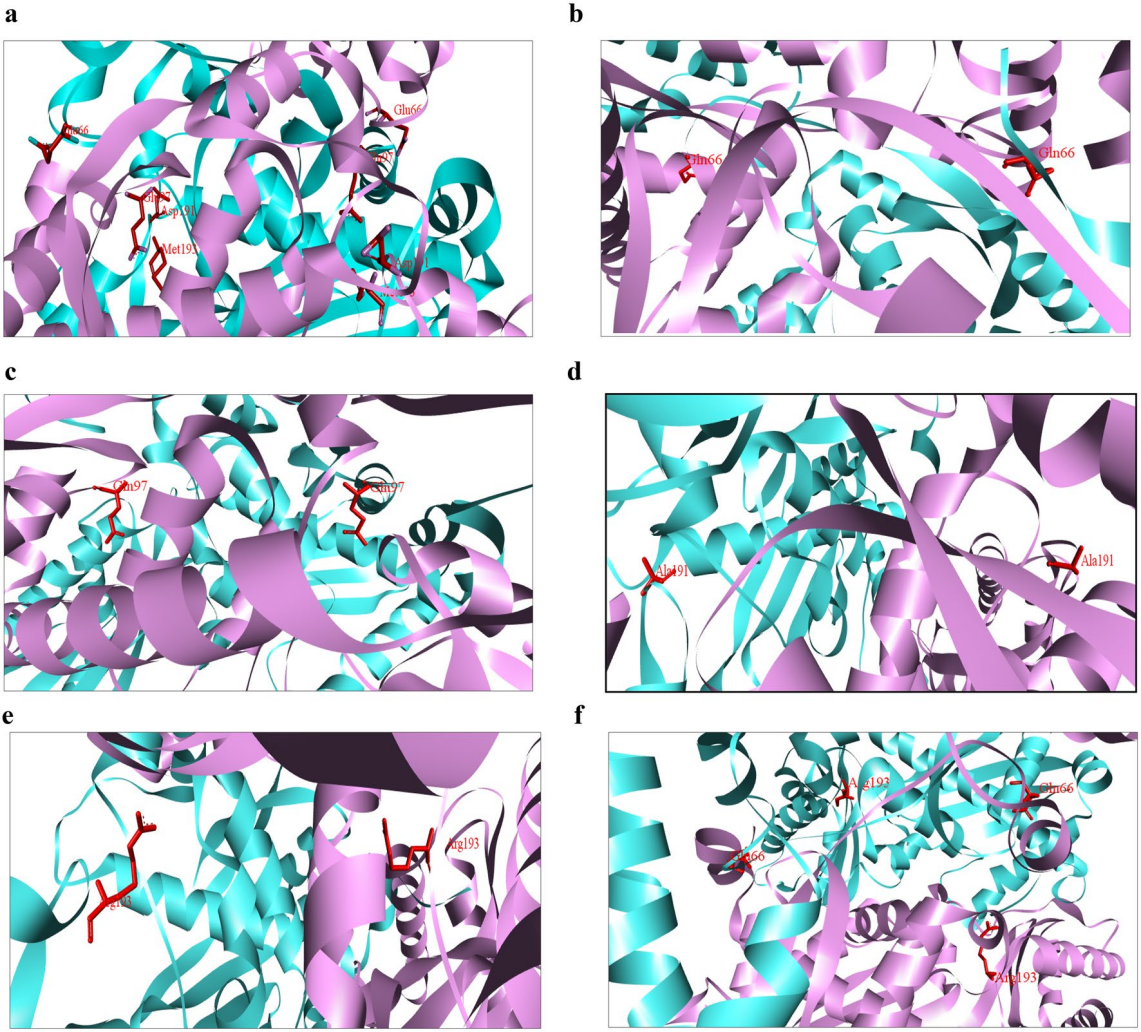
Structural modeling of the conformational differences between wild-type and mutant HMGR proteins of *Candida glabrata* (*Cg*HMGR), in flat ribbon representation. (**a**) Wild-type *Cg*HMGR, (**b**) mutant *Cg*HMGRE680Q, (**c**) mutant *Cg*HMGRE711Q, (**d**) mutant *Cg*HMGRD805A, (**e**) mutant *Cg*HMGRM807R, and (**f**) mutant *Cg*HMGRE680Q-M807R. The α monomer is illustrated in pink, the β monomer in cyan, and the mutation in stick representation (red). Made using Discovery Studio software.

The homology modeling of the five mutants herein generated was carried out in the Modeller program, using the crystal structure of *h*HMGR (PDB: 1DQA) as the template. Of the fifteen models of each mutant provided, the one with the lowest DOPE score (the most thermodynamically stable) was selected. The selected models are portrayed in Fig. 3, highlighting in red the amino acids that were changed in the transition from the wild-type (Fig. 3a) to the mutant proteins (Fig. 3b–f): for E680Q, Glu66 to Gln66; for E711Q, Glu97 to Gln97; for D805A, Asp191 to Ala191; and for M807R, Met193 to Arg193.

The models were validated with the PROCHECK program, which quantifies the loss of residues in the allowed regions of a Ramachandran plot (Supplementary Fig. S2). In all the models, over 90% of the residues are found in the most favorable regions, with no residues in the disallowed regions (Supplementary Table 1). All the models constructed had Ramachandran statistics similar to those reported in the wild-type *Cg*HMGR model. These results confirmed the high quality and reliability of the models generated.

### Docking study of ligand binding to wild-type and mutant HMGR proteins

An *in-silico* analysis was carried out for the interaction of mutant and wild-type *Cg*HMGR proteins with two of their inhibitors (simvastatin and alpha-asarone) and their substrate (HMG-CoA) (Fig. S5). Each mutant showed a lower binding energy for each ligand than the wild-type *Cg*HMGR (Fig. S5, Table 2). The mutant protein E711Q displayed the lowest value (Fig. S5). Glu97, Asp307, Lys321 and His403 are the residues most frequently involved in binding, being located in the catalytic domain of *Cg*HMGR. However, not all of these amino acids participate in the binding of the ligands with each of the mutant peptides (Table 2).

Regarding the binding of simvastatin, alpha-asarone, and HMG-CoA to the active site of the *Cg*HMGR enzyme, the docking simulations gave insights into the binding energies as well as the residues, polar interactions, and hydrophobic interactions involved (Table 1).

**Table 1:** Results of docking simvastatin, alpha asarone, and HMG-CoA at the active site of HMGR of *Candida glabrata* (*Cg*HMGR).

Protein	Compound	Binding energy ΔG (kcal/mol)	Residues interacting with the ligand	Polar interactions	Hydro-phobic interactions
Wild-type *Cg*HMGR	Simvastatin	− 10.71	Glu97, Met193, Asp307	Glu97, Met193, Asp307	Met193
	Alpha-asarone	− 6.4	Thr96, Glu9, Ala192, Met193, Met197, Gly305, Gln306, Gln310, Thr346	Glu97, Gln306,Thr346	Ala192, Met197
	HMG-CoA	− 6.48	Asp128, Lys231, Lys275, Asn407	Lys231, Lys275, Asn407	Asp128
*Cg*HMGR E680Q	Simvastatin	− 10.18	Ala192, Met193, Asn196, Asp307, Gly344, Gy345, His403	Asn196, Asp307	Ala192, His403
	Alpha-asarone	− 5.81	Asp230, Lys231, Lys275, Asn290, Ser315	Asp230, Asn290, Ser315	Lys275
	HMG-CoA	− 6.31	Arg128, Ser224, Asp230, Lys231, Lys232, Lys275	Ser224, Asp230	Lys232, Lys275
*Cg*HMGR E711Q	Simvastatin	− 7.1	Gly98, Arg128, Lys231, His292, Asn295, Leu390, Leu394, Leu399	Gly98, Arg128, His292, Asn295, Leu399, His403	Lys231, Leu390
	Alpha-asarone	− 5.7	Lys231, Lys232, Pro233, Gly288, Ala294, Ser315, Asn316	Gly288, Ser315	Lys232, Pro233, Ala294, Asn316
	HMG-CoA	− 6.05	Arg128, Ser224, Asn226, Lys232, Lys275	Ser224, Lys232, Lys275	Arg128, Lys232
*Cg*HMGR D805A	Simvastatin	− 9.54	Leu74, Leu302, Asp307	Asp307	Leu74, Leu302
	Alpha-asarone	− 6.33	Arg128, Ser199, Leu399, His403, Met404, Val498	Arg128, Ser199	Leu399, His403, Val498
	HMG-CoA	− 6.26	Asn196, Ser199, Lys200, Glu203, His403, Arg408	Glu203, Ser199, His403	Glu203, His403
*Cg*HMGR M807R	Simvastatin	− 9.78	Glu97, Arg128, Val223, Asp230, Asn295, Leu399, Val400, His403	Glu97, Asp230	Val223, Val400
	Alpha-asarone	− 6.86	Glu97, Lys231, Lys232, Ala293, Ala294, Asn295	Lys232	Glu97, Lys232, Ala293, Ala294, Asn295
	HMG-CoA	− 6.35	Arg106, Leu399, His403	Arg106, Leu399, His403	–
*Cg*HMGR E680Q-M807R	Simvastatin	− 7.36	Ala63, Cys64, Glu97, Asp307, Arg408	Glu97, Asp307	Ala63, Cys64, Arg408
	Alpha-asarone	− 5.8	Lys231, Pro233, Val286, Ala294	–	Lys231, Pro233, Val286, Ala294
	HMG-CoA	− 6.13	Cys65, Val68, Ile69, Tyr71, Thr95, Thr96	Cys65, Val68, Ile69, Tyr71, Thr95, Thr96	–

### Generation and verification of the point mutants of HMGR genes

PCR reactions were carried out with specific oligonucleotides designed to obtain the following mutants: *Cg*HMGRE680Q, *Cg*HMGRE711Q, *Cg*HMGRD805A, *Cg*HMGRM807R and *Cg*HMGRE680Q-M807R. The plasmid Rec-MBP-*Cg*HMGR was used as the template DNA (where MBP refers to the maltose-binding protein). The amplified oligonucleotides had a size of 7.9 kbp (Supplementary Fig. S3a), which corresponds to the size of the plasmid pMAL-C2X (6.6 kbp) plus the gene encoding the soluble fraction of *Cg*HMGR (1.3 kbp). The full-length gel original is included in Supplementary Information (Fig. S3c). Additionally, the plasmids afforded by the transformation of each of the amplified products were subjected to double digestion (Supplementary Fig. S3b), finding the expected molecules of 6.6 and 1.3 kbp. The full-length blot is also in Supplementary Information (Fig. S3d).

Likewise, the mutations generated by sequencing were corroborated. The sequences obtained were aligned with the coding sequence of wild-type *Cg*HMGR to confirm that the expected mutations had indeed been introduced. For *Cg*HMGRE680Q and *Cg*HMGRE711Q, the triplet changed from GAA to CAA, though in a distinct position in each case. The GAT triplet changed to GCT in *Cg*HMGRD805A, and CAT to CCT in *Cg*HMGRM807R. For the double mutant *Cg*HMGRE680Q-M807R, TTC changed to TTG and CAT to CCT (Fig. S6). The sequencing revealed that the point mutations were introduced successfully.

### Expression, detection, and purification of CgHMGR mutant proteins

**Figure 4:**
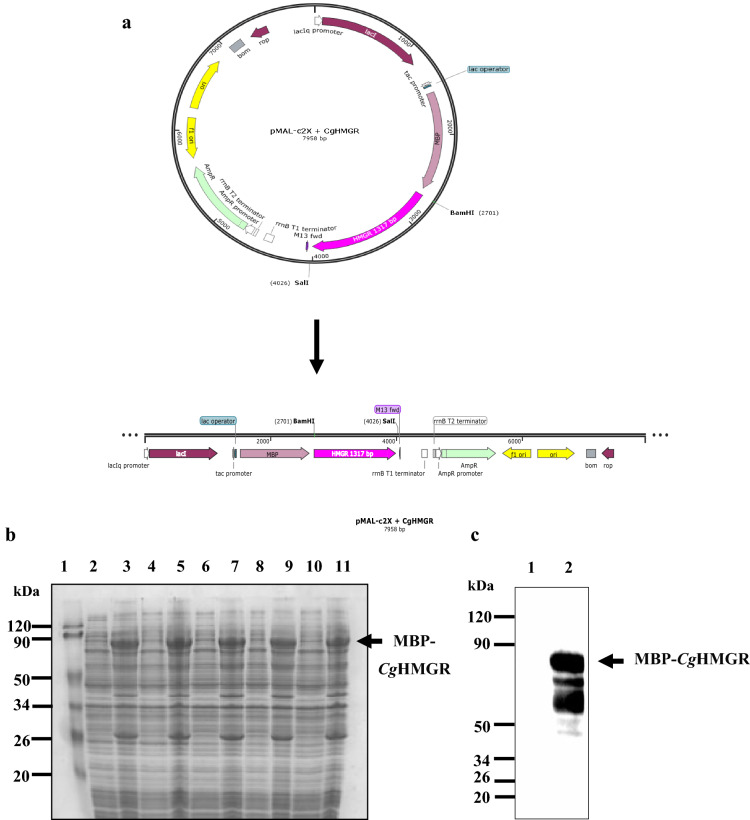
Graphic representation, overexpression, and detection of the recombinant *Cg*HMGRE680Q, *Cg*HMGRE711Q, *Cg*HMGR805A, *Cg*HMGRM807R and *Cg*HMGRE680Q-M807R proteins. (**a**) Cartoon illustration of the p-MBP-*Cg*HMGR (where MBP refers to the maltose-binding protein), which was used to obtain and express mutant *Cg*HMGR proteins. (**b**) SDS-PAGE of the expression of the mutated proteins: Lane 1, molecular weight marker; lanes 2, 4, 6, 8 and 10, extract of cells before induction; lanes 3, 5, 7, 9 and 11, extract of cells after induction with IPTG. The full-length gel is included in Supplementary Information (Fig. S4a). (**c**) Example of the verification of protein identity by Western blot based on anti-MBP antibodies: Lane 1, molecular weight marker; lane 2, uninduced cell extract; lane 3, induced cell extract. The full-length blot is also shown in Supplementary Information (Fig. S4b).

The protein expression of the *Cg*HMGRE680Q, *Cg*HMGRE711Q, *Cg*HMGRD805A, *Cg*HMGRM807R and *Cg*HMGRE680Q-M807R mutants was induced with IPTG. Samples taken before and after induction were visualized with SDS-PAGE. The gel in Fig. 4b shows the overexpression of the proteins of interest. The approximate molecular mass of 90 kDa corresponds to the fusion of *Cg*HMGR + MBP (Fig. 4a). The pMAL vector favored the expression of the recombinant proteins, which fused to the soluble MBP. The latter facilitated the isolation of the enzyme after its overexpression.

Hence, the mutants were overexpressed and the presence of MBP fused to the mutants was verified. The mutants found were the same as those previously identified by using anti-MBP antibodies (Fig. 4c). Subsequently, the mutants were purified easily (in a few steps) by means of amylose affinity chromatography. In all cases, a single band appeared at the approximate molecular mass of 90 kDa, exhibiting the same behavior as wild-type *Cg*HMGR.

It was demonstrated by a coupling study that the substrate (HMG-CoA) and probable inhibitors (simvastatin and alpha-asarone) of *Cg*HMGR did not recognize a specific site in MBP. Based on the resulting low binding energy values, ​​these compounds seem to have very limited affinity towards MBP. Moreover, HMG-CoA selectively binds to HMGR, as has been observed for *h*HMGR. The active site and the residues involved in catalysis have been reported (Andrade-Pavón et al., 2017). Since *Cg*HMGR displayed strong enzymatic activity, and the clone of *E. coli* with an empty plasmid did not show HMGR activity, the presence of MBP did not affect the activity of this enzyme^10^.

### Reduced enzymatic activity of the mutants compared to wild-type CgHMGR

The enzymatic activity of the *Cg*HMGRE680Q, *Cg*HMGRE711Q, *Cg*HMGRD805A, *Cg*HMGRM807R and *Cg*HMGRE680Q-M807R proteins was measured spectrophotometrically by reading the decrease in absorbance of NADPH at 340 nm during 10 min. For each mutant, this parameter was determined in triplicate and expressed as the percentage of activity of the wild-type protein (considered as 100%) (Table 2).

**Table 2:** The enzymatic activity and binding energies of the point mutations made in the motifs of the dimerization site, substrate binding site, and cofactor binding site of *Cg*HMGR.

Rec-*Cg*HMGR proteins	Point mutations, affected motif	Simva-statin	Alpha-asarone	HMG-CoA	Specific enzymatic activity	Percentage of HMGR activity
		Binding free energy (Kcal/mol)			(mU/mg of protein)	(% ± SD)
*Cg*HMGR-wild-type		− 10.71	− 6.4	− 6.48	13.6	100
*Cg*HMGR- E680Q Glu x Gln	Dimer-ization ENVIG QNVIG	− 10.18	− 5.81	− 6.31	11.2	82.2 ± 7.8***
*Cg*HMGR- E711Q Glu x Gln	Substrate binding EGCLVAS QGCLVAS	− 7.14	− 5.7	− 6.05	3.1	23.4 ± 7.8***
*Cg*HMGR- D805A Asp x Ala	Cofactor binding DAMGMN AAMGMN	− 9.54	− 6.33	− 6.26	7.0	51.7 ± 1.6***
*Cg*HMGR-M807R Met x Arg	Cofactor binding DAMGMN DARGMN	− 9.78	− 6.86	− 6.35	10.7	79 ± 2.1**
*Cg*HMGR-E680Q-M807R Glu x Gln, Met x Arg	Dimer-ization ENVIG QNVIG Cofactor binding DAMGMN DARGMN	− 7.36	− 5.8	− 6.13	5.9	44 ± 9.8*

Point mutations were made in the catalytic domain of *Cg*HMGR to express various recombinant proteins (Rec-*Cg*HMGR) (as described in Material and Methods). The aa of the wild-type sequence is shown in green and that of the mutated protein in red. The site of the motif sequence is indicated. The binding energies of *Cg*HMGR (wild-type and mutated) with its HMG-CoA substrate and probable inhibitors (simvastatin and alpha-asarone) were estimated by docking. The enzymatic activity of the recombinant enzymes is calculated as a percentage of the activity of the wild-type enzyme (considered as 100%). Data are expressed as the average of three independent replicates ± standard deviation (SD). ***P < 0.0005 **P < 0.0052, *P < 0.0153. Significant differences compared to the wild-type protein based on a paired Student's *t*-type analysis.

Compared to wild-type *Cg*HMGR, all five mutants displayed a lower enzymatic activity. The smallest percentage of residual activity (23%) was observed for E711Q. The least affected enzyme was E680Q (mutated at the dimerization site), with 88.2% residual activity. The double mutant E680Q-M807R exhibited the second lowest activity (44%), while the residual activity of the corresponding single mutants, E680Q and M807R, was substantially higher (82% and 79%, respectively). The enzymatic activity of each mutant was significantly less than that of the wild-type protein, according to a paired Student's *t*-type analysis.

### Kinetic parameters of the mutants

For the determination of kinetic parameters (*K*_*M*_ and *V*_max)_, activity was assayed in a reaction mixture containing increasing amounts of HMG-CoA, ranging from 1 to 64 μM. Enzymatic activity was obtained from monitoring the conversion of HMGCoA-dependent oxidation of NADPH and taking the linear fraction for each of the substrate concentrations to obtain a Michaelis–Menten plot (Fig. S7). The data were evaluated with the Lineweaver–Burk and Hanes-Woolf models (Fig. S7). The Hanes-Woolf model was used because of explaining the results better than the other. Moreover, the Hanes-Woolf data analysis led to an outcome comparable to that described in a previous report on the wild-type protein^10^. The present data for the *K*_*M*_ and *V*_*max*_ was as follows: 8.31 µM and 0.28 µM min^−1^ for *Cg*HMGRE680Q, 18 µM and 0.70 µM min^−1^ for *Cg*HMGRE711Q, and 11.84 µM and 0.43 µM min^−1^ for *Cg*HMGRD805A.

## Discussion

HMGR is a highly conserved enzyme anchored to the endoplasmic reticulum membrane of eukaryotes and to the plasma membrane of prokaryotes. It is responsible for the synthesis of cholesterol in mammals, ergosterol in fungi, and isoprenoids in bacteria^1^. Intense research efforts have focused on *h*HMGR as the target of inhibitors such as statins and fibrates, which diminish the synthesis of cholesterol and reduce the risk of cardiac arrest in patients with hyperlipidemia. Consequently, *h*HMGR has been examined at many levels: transcription, translational regulation, synthesis, inhibition, crystallization, and tertiary structure. However, reports on point mutations in non-human HMGR are limited, to the best of our knowledge, to *P. mevalonii* and Syrian hamsters. The current investigation is the first to make point mutations in the HMGR enzyme of a yeast of medical interest.

The alignment presently carried out with the amino acid sequences of HMGRs from various species demonstrated that the motif sequences of the dimerization site (ENVIG), the substrate binding site (EGCLVAS), and the cofactor binding site (DAMGMN) are highly conserved, regardless of whether they are from fungal, mammalian, plant or bacterial proteins. Hence, these amino acid residues appear to have an essential function in the catalytic reaction of the enzyme and/or in its conformation and tertiary structure, which makes them candidates for the evaluation of point mutants in key sequences.

On the other hand, the phylogenetic analysis of the HMGR proteins from different yeasts revealed a classification in accordance with their taxonomy. The majority are of the Phylum Ascomycota, which includes the *Candida* and *Saccharomyces* genera. *Cg*HMGR is grouped with the yeast proteins of the WGD clade, encompassing *K. lactis* and *S. cerevisiae*^17,18^, and not with those of the CTG clade, containing *C. albicans* and other *Candida* species. Therefore, *C. glabrata* is a species phylogenetically closer to *S. cerevisiae* than to *C. albicans*. The HMGRs of *Y. lipolytica, S. pombe* and *U. maydis* (the latter being a fungus of the phylum Basidiomycota) belong to branches completely independent of Ascomycota. The HMGRs of other eukaryotes (e.g., plants, insects and mammals) are grouped into an independent clade and the HMGR of the bacterium *P. mevalonii* functions as an external group, further validating the previous results^1^.

*h*HMGR, purified and crystallized in the year 2000, was used as a template for the molecular modeling of proteins^19^. Although the three-dimensional structure of the catalytic domain of *h*HMGR (426–888 aa) is a tetramer, it has been suggested that the protein dimer might be able to bind to the HMG-CoA substrate^19^. The molecular modeling of the five *Cg*HMGR with point mutations generated proteins made up of two identical subunits (dimers). The structures of the mutated proteins provided an a priori approximation to the 3D structure of the corresponding peptides, a necessary step for their in-silico analysis^11^.

According to kinetic studies on *h*HMGR, statins compete for the HMG-CoA substrate without affecting binding to the NADPH cofactor^20^. Hence, the substitution of an amino acid residue in the conserved sequences could plausibly alter the affinity of the enzyme for its substrate HMG-CoA as well as for simvastatin and alpha-asarone. To explore such a possibility, a molecular docking study was carried out.

This is the first report, to our knowledge, of an evaluation of molecular coupling between mutant proteins of *Cg*HMGR and reference inhibitors. Information is herein provided on the substantial effect of mutations to specific amino acids on the ligand-receptor interaction.

The amino acids Glu97, Asp307, Lys321 and His403 are part of the catalytic domain of *Cg*HMGR and their plausible role in the ligand-HMGR interaction has been proposed^1,11^. According to previous docking studies, the amino acids affected by the mutations likely influence the recognition of the substrate/inhibitor and/or alter the native structure of the protein^21^.

Statins and alpha-asarone are competitive inhibitors of HMGRs, blocking access to the HMG-CoA substrate^1,3,10,11^. The *Cg*HMGR mutant in the substrate binding motif, E711Q, displayed the lowest binding energy for the ligands (simvastatin, alpha-asarone, and the HMG-CoA substrate) compared to the other mutants herein examined. The limited enzymatic activity and binding energy ​​of this mutant was expected, considering that the three ligands currently tested interact with the EGCLVAS motif (of the substrate binding site)^3,10,11^. Glutamic acid in the EGCLVAS region seems to have an important function in the catalysis of *Cg*HMGR, as previously reported^12^.

*Cg*HMGR-M807R was mutated at the cofactor binding site, specifically at the methionine position 807 of the DAMGMN sequence^12^. The change was from methionine, an amino acid with a neutral and nonpolar charge, to arginine, one with a positive charge. Although both amino acids participate in protein methylation^22^, the positive charge of arginine could be detrimental to the binding of the cofactor to the enzyme. Additionally, arginine contains a guanidinium group, which when ionized has a lower charge density than other amino acids. While methionine is a weak nucleophile and cannot be protonated^22^, the positive charge of arginine might be able to alter the α-helix or β-folded structure of the protein and consequently its enzymatic activity. Finally, the larger size of arginine than methionine may modify the structure of the protein. It is possible that these factors hindered the proper binding of the enzyme to its cofactor, which would contribute to the reduced enzymatic activity.

The mutation made in the dimerization site caused glutamic acid to be replaced by glutamine in position 680. Although both amino acids are very similar in size, glutamic acid has a negative charge and glutamine a polar neutral charge. Glutamic acid has carboxylate (COO–) side chains that are potential proton acceptors, forming hydrogen bonds and thus the secondary structure of the protein (β-folded sheets or α-helix)^23^. This amino acid could possibly have a similar function as the Glu83 residue, which is highly conserved and participates in catalysis by transferring protons to Lys267. When glutamic acid carries out the decomposition of mevaldyl-CoA, its protonation and deprotonation confers distinct levels of energy to the reaction, and these are necessary for the enzymatic activity that gives rise to sterols^24^. Glutamine is a glutamic acid amide afforded by the replacement of the hydroxyl of glutamic acid with an amine group^24^. Since it has a polar neutral charge, however, it might have affected the interaction with the amino acids belonging to the ENVIG sequence. Therefore, the proper formation of the dimers may not have taken place, which would lead to the inadequate binding of the enzyme to the cofactor and consequently to limited enzymatic activity.

It is not surprising that the double mutant (*Cg*HMGRE680Q-M807R) exhibited the second lowest enzymatic activity of the present mutants. The two mutations occurred at sites where amino acids are highly conserved: the sequence of the cofactor binding site (DAMGMN) and of the dimerization site (ENVIG).

There was a greater *K*_*M*_ value found for each mutant than for the wild-type protein^10^. Hence, the data suggest that the affinity of HMG-CoA would be lower for the mutant versions than for the wild-type protein. Indeed, a marked decrease was detected in the enzymatic activity of the mutants herein examined compared to that of the wild-type protein.

*C. glabrata* has only one *HMG1* gene, which is orthologous to the *S. cerevisiae* gene^7^. In the current contribution, the sensitivity of *Cg*HMGR mutants to antifungals was not evaluated. According to a previous study, the mutation of this gene in *Aspergillus fumigatus* causes resistance to antifungal compounds of the triazole class^25,26^. The mutation of the enzymes HMG1 and HMG2 of *S. cerevisiae* leads to a great sensitivity to compactin, an HMGR inhibitor^27^. There are no reports, to our knowledge, on a double mutant *cdr1∆ hmg1∆*. However, these mutants have been assessed separately, observing an increase in sensitivity to azoles caused by *cdr1∆* in *C. glabrata* as well as resistance to the same drugs produced by *hmg1∆* in *Aspergillus fumigatus*^25,26,28^.

The insights gained from the presently mutated proteins can be applied not only to the improvement of *C. glabrata* as a model for research on drug resistance, but also to the investigation of the HMGR of additional species, such as *C. auris* and *C. haemulonii*, which have recently emerged as pathogenic multi-drug resistant strains^29^. The emergence of *Candida* infections associated with SARS-Cov2 gives greater emphasis to the urgency of developing antifungal agents for alternative targets^30^.

## Conclusions

Five point mutants were made at three highly conserved sites of the catalytic domain of HMGR: the dimerization, substrate binding and cofactor binding sites. After the mutants were expressed, they were detected and purified to verify their integrity. All the mutants exhibited significantly lower enzymatic activity and ligand affinity than wild-type *Cg*HMGR. The in vitro and in silico testing of the mutants gave insights into the function of specific amino acid residues in the catalytic site of the enzyme, especially glutamic acid. The current findings should certainly facilitate the creation of new mutants of HMGR to further explore the catalysis, recognition, conformation, and dimerization mechanisms involved in the activity of the enzyme and the viability of *C. glabrata* and other opportunistic pathogenic fungi.

## Materials and methods

### Multiple sequence alignment of HMGR enzymes from different organisms

The amino acid sequences were downloaded from the NCBI database (https://www.ncbi.nlm.nih.gov/) for the HMGR enzymes of the following organisms: *P. mevalonii* (WP_016714288), *Arabidopsis thaliana* (NP_177775), *D. melanogaster* (NP_732900), *H. sapiens* (XP_011541659), *Mus musculus* (NP_001347095), *Rattus norvegicus* (NP_037266), *U. maydis* 521 (XP_011389590), *S. pombe* (NP_588235), *Y. lipolytica* CLIB122 (XP_503558), *C. auris* (XP_028890531), *C. haemulonii* (XP_025340375.1)*, C. lusitaniae* ATCC 42,720 (XP_002615608), *M. guilliermondii* ATCC 6260 (XP_001482757), *D. hansenii* CBS767 (also called *Candida famata*) (XP_458872), *C. parapsilosis* (KAF6043359), *C. orthopsilosis* Co 90–125 (XP_003868277), *C. tropicalis* MYA-3404 (XP_002550050), *C. albicans* SC5314 (XP_713636), *C. dubliniensis* CD36 (XP_002417024), *C. kefyr* (QGN16198), *K. lactis* NRRL Y-1140 (XP_451740), *C. glabrata* CBS138 (XP_449268), *S. cerevisiae* S288c HMG1(NP_013636) and *S. cerevisiae* 2288c HMG2 (NP_013555)^31^. Once the sequences corresponding to the catalytic domain of such enzymes were downloaded, they were selected and subjected to multiple alignment in the Clustal Omega program^32^. The motif domains (structural motifs) were located with the ExPASy server, PROSITE (https://prosite.expasy.org/)^33^, and visualized with the web server WebLogo (http://weblogo.berkeley.edu/logo.cgi).

### Phylogenetic analysis of HMGR proteins from different organisms

A phylogenetic tree was constructed with the sequences of the catalytic domain of the aforementioned twenty-four HMGRs in the MEGA6 program^34^, utilizing the maximum likelihood method and the WAG + G model. Additionally, a bootstrap replication of 100 was used to evaluate the reliability of the phylogenetic tree.

### Homology modeling of CgHMGR mutant proteins

Fifteen models were generated for each of the five *Cg*HMGR mutant proteins with Modeller 9.24^35^. The models with the lowest discrete optimized protein energy (DOPE) score were selected and edited in Discovery Studio^36^, then subjected to a validation process to assess their stereochemical quality with the PROCHECK^37^ tool integrated in the Structural Analysis and Verification Server (SAVES) (https://servicesn.mbi.ucla.edu/SAVES/). This tool also quantified the amino acid residues in the available regions of the Ramachandran plots.

### Docking studies

The ligands were downloaded from the PubChem server^38^ in 2D format and transformed into 3D mol2 format in the Open Babel GUI program^39^. Kollman charges were assigned to the hydrogen atoms of the protein. Preparation of the docking parameters and molecular coupling of the five mutants to the ligands (HMG-CoA, simvastatin, and alpha asarone) was carried out on AutoDock Tool 4.0^40^. The grid dimensions were set at 128 × 88 × 64 Å^3^ with points separated by 0.375 Å, and the grid center was X = − 10.517, Y = 14.058 and Z = 22.162. One hundred docking runs were made with the Lamarckian genetic algorithm and various parameters were estimated. The results were compared between ligands based on their lowest free coupling energy. Interactions were visualized with Discovery Studio.

### Microorganisms and plasmids

Transformation was achieved with the *Escherichia coli* DH10β strain and the overexpression of heterologous proteins with the *E. coli* BL21 strain. The plasmid pMAL-C2X-*Cg*HMGR, which was previously obtained by our group and harbors the gene encoding the wild-type protein, served as the template DNA for generating the mutants^10^*.*

### Culture media

*E. coli* harboring the plasmids was grown in liquid Luria Bertani (LB) medium (1.0% casein peptone, 0.5% yeast extract, and 0.5% NaCl) and solid LB medium with 1.5% bacteriological agar. These media were adjusted to pH 7.0 and supplemented with 100 μg/mL ampicillin. Culture media and antibiotics were acquired from Sigma Aldrich (St. Louis, MO, USA).

### Mutagenic oligonucleotide design

The mutagenic primers were designed manually, taking as templates the sequences of the dimerization site, the substrate binding site, and the cofactor binding site within the catalytic domain of the HMGR enzyme. Consideration was given to 18 base pairs to the left and 18 base pairs to the right of the codon where the mutation was designed, resulting in a total of 39 nucleotides for each primer. Point mutations were made involving the following changes: glutamic acid to glutamine in the dimerization site (ENVIG) to provide E680Q, aspartic acid to glutamine in the substrate binding site (EGCLVAS) to afford E711Q, aspartic acid to alanine in the cofactor binding site (DAMGMN) to furnish (D805A), and methionine to arginine in the cofactor binding site to yield M807R. The double mutation of E680Q-M807R was formed by the respective single point mutations.

The oligonucleotides herein designed were synthesized at the Institute of Biotechnology of the National Autonomous University of Mexico (UNAM). The melting temperature of the oligonucleotides was calculated after their synthesis, using the bioinformatics program Oligo Analyzer from Integrated DNA Technologies (IDT) (https://www.idtdna.com/pages/tools/oligoanalyzer). Table 3 shows the original sequence (wild-type *Cg*HMGR) and the mutagenic oligonucleotides. The codon of the amino acid to be mutated is illustrated in red, and the change made is underlined.

**Table 3:** Oligonucleotides designed to obtain the following point mutants: E680Q, E711Q, D805A, M807R and E680Q-M807R.

Mutated aa	Direction	39 pb Oligonucleotide sequences	T_m_	%GC
E680	Fw	5′ GTCTTTGGTGCTTGTTGTGAAAATGTGATTGGTTACATG 3′	75.4	62.9
E680Q	Fw	5′ GTCTTTGGTGCTTGTTGTCAAAATGTGATTGGTTACATG 3′	75.4	62.9
E680Q	Rv	5′ CATGTAACCAATCACATTTTGACAACAAGCACCAAAGAC3′	75.4	62.9
E711	Fw	5′ ATCCCTATGGCCACAACTGAAGGTTGCTTGGTTGCATCT3′	80.1	67.1
E711Q	Fw	5′ ATCCCTATGGCCACAACTCAAGGTTGCTTGGTTGCATCT 3′	80.1	67.1
E711Q	Rv	5′AGATGCAACCAAGCAACCTTGAGTTGTGGCCATAGGGAT 3′	80.1	67.1
D805	Fw	5′ TTTAGAACTACAACTGGTGATGCGATGGGTATGAATATG 3′	73.0	62.9
D805A	Fw	5′ TTTAGAACTACAACTGGTGCTGCGATGGGTATGAATATG 3′	74.2	63.9
D805A	Rv	5′ CATATTCATACCCATCGCAGCACCAGTTGTAGTTCTAAA 3′	74.2	63.9
M807	Fw	5′ACTACAACTGGTGATGCGATGGGTATGAATATGATTTCT3′	73.5	62.9
M807R	Fw	5′ACTACAACTGGTGATGCGAGGGGTATGAATATGATTTCT3′	74.1	63.9
M807R	Rv	5′AGAAATCATATTCATACCCCTCGCATCACCAGTTGTAGT3′	74.1	63.9

T_m_: melting temperature; %GC: Guanine and Cytosine Percentage.

### Generation of CgHMGR point mutants by PCR

The E680Q, E711Q and D805A point mutants were generated by PCR with a "Quick Change" commercial kit (Thermo Fisher Scientific, USA), as specified in the manufacturer’s instructions with some modifications. This procedure is based on a PCR reaction with two complementary and antiparallel oligonucleotides and the appropriate DNA polymerase to achieve the desired mutation. Each oligonucleotide hybridizes with one of the two strands of the plasmid to be amplified. The plasmid obtained led to the sequence to be mutated (pMAL-c2X-*Cg*HMGR), which was used as the template DNA. Mutants M807R and E680Q-M807R were produced by inverse PCR, with plasmid pMBP-*Cg*HMGR as the template DNA^10,11^. For the double mutant, the plasmid DNA of the rec-*Cg*HMGR-E680Q mutant and the mutagenic oligonucleotide M807R served as the template DNA.

Once the reaction conditions were established and developed, the amplicon was digested with the Dpn-I enzyme (Thermo Fisher Scientific, USA). *E. coli* DH10β cells were then transformed with the digested PCR product. Subsequently, *E. coli* cells were plated on LB agar with ampicillin (100 µg/ml) and incubated at 37 °C for 24 h. The transforming colonies were selected for the extraction of plasmid DNA. Plasmids were purified with the ZymoPURE plasmid Miniprep Kit (Zymo Research, CA, USA) and confirmed by sequencing them according to the Sanger method, a procedure that demonstrated the presence of the desired mutations (Macrogen®, Seoul Korea).

### Expression, purification and detection of wild-type and mutant recombinant proteins

Once the presence of the desired mutations was confirmed by sequencing the corresponding plasmids, the recombinant proteins were expressed. Briefly, *E. coli* BL21 cells were transformed with the mutated DNA by growing the cells containing the fusion plasmid in LB medium plus ampicillin (100 µg/mL) and 0.2% glucose until reaching an optical density of 0.6 at 600 nm. IPTG was added immediately (0.3 mM final concentration) to induce the expression of the protein, a process carried out at 37 °C for 4 h. Upon completion of this time, cells were harvested at 4000 × g for 20 min at 4 °C. The cells were mechanically broken by using glass beads and a vortex, and then the cell pack was dissolved in a column buffer (1 M Tris–HCl, pH 7.4, 11.7 g NaCl, 0.5 M EDTA, and 154 mg DTT) to obtain a cell-free extract. The cells were disrupted for 20 min, alternating every other 30 s on and off ice. The cell lysate was centrifuged at 18,000 g for 20 min and the supernatant (cell-free extract) was recovered. The protein was purified by affinity column chromatography with amylose resin (New England Biolabs, USA). The wild-type and mutant proteins were eluted with a buffer supplemented with 10 mM maltose. Fractions were collected to determine *Cg*HMGR activity and protein concentration by the Lowry method. The expression and the successful purification of the mutant proteins was visualized by means of 10% SDS-PAGE stained with Coomassie blue. The mutated proteins were detected by Western blot with the anti-MBP monoclonal antibody (New England Biolabs, USA), according to the manufacturer’s specifications.

### The HMGR enzymatic activity assay

The enzymatic activity of the five mutants was quantified by the method of Bischoff and Rodwell^41^, beginning with the elaboration of a reaction mixture containing 10 mM HMG-CoA, 50 mM Tris–HCl regulator, injectable water, 9 µg of the protein of the respective mutant, and 10 mM NADPH. The cofactor (NADPH) is oxidized by the catalytic subunit of HMGR in the presence of the substrate HMG-CoA, causing a decrease in absorbance. Thus, absorbance was monitored at 340 nm for 10 min. Reagents were purchased from Sigma Aldrich (St Louis, MO, USA). One unit of specific activity was defined as the amount of enzyme that oxidizes 1 µmol of NADPH to NADP + per minute per milligram of protein. All spectrophotometric readings were made on a BioSpectrometer® kinetic apparatus (Eppendorf AG, Germany).

### Substrate specificity and kinetic parameters

The steady-state kinetic constants for mutants were established by means of the standard activity assay under the same conditions described in the previous section. Activity was determined by incubating reaction mixtures with substrate concentrations ranging from 1 to 64 µM. The *K*_*M*_ and *V*_*max*_ constants were obtained from Hanes-Woolf plots and expressed as the mean of three different experiments^10,42^.

## Supplementary Information

Supplementary Information.
