# Supplementary material for: Point mutations in Candida glabrata 3-hydroxy-3-methylglutaryl-coenzyme A reductase (CgHMGR) decrease enzymatic activity and substrate/inhibitor affinity
Source: Sci Rep. 2021 Oct 21;11:20842. doi: 10.1038/s41598-021-00356-w (PMC8531335; doi:10.1038/s41598-021-00356-w)
Supplement: Supplementary file 1 — Supplementary Information. [file 41598_2021_356_MOESM1_ESM.pdf]

Supplementary Information

Point mutations in *Candida glabrata* 3-hydroxy-3-methylglutaryl-coenzyme A reductase (*Cg*HMGR) decrease enzymatic activity and substrate/inhibitor affinity

Dulce Andrade-Pavón[1], Vanessa Fernández-Muñoz[1], Wendy González-Ibarra[2], César Hernández-Rodríguez[1], J. Antonio Ibarra[2], and Lourdes Villa-Tanaca[1,]*

[1]Laboratorio de Biología Molecular de Bacterias y Levaduras. Departamento de Microbiología, Escuela Nacional de Ciencias Biológicas, Instituto Politécnico Nacional, CDMX. [2]Laboratorio de Genética Microbiana. Departamento de Microbiología, Escuela Nacional de Ciencias Biológicas, Instituto Politécnico Nacional, CDMX.

*Corresponding author:
Lourdes Villa-Tanaca. Laboratorio de Biología Molecular de Bacterias y Levaduras. Departamento de Microbiología, Escuela Nacional de Ciencias Biológicas, Instituto Politécnico Nacional, Prol. de Carpio y Plan de Ayala. Col. Sto. Tomás, México, Distrito Federal, CP 11340, México. Phone 52+55 57296000 ext. 62569. Email: mvillat@ipn.mx; lourdesvillatanka@gmail.com

```
Pseudomonas mevalonii        ------MSLDS--RLPAFRNLSP--------AARLDHIGQLLGLSHDDVSLLANAGALPM 44
Arabidopsis thaliana         DEEIVKSVIDG--VIPSYSLESRLGDCKRAASIRREALQRVTGR---SIEGLPLDGFDYE 55
Drosophila melanogaster      DEEIVSIVHAGgtHCPLHKIESVLDDPERGVRIRRQIIGSRAKMPvgrLDVLPYEHFDYR 60
Homo sapiens                 DAEIIQLVNAK--HIPAYKLETLMETHERGVSIRRQLLSKKLSEPs-sLQYLPYRDYNYS 57
Mus musculus                 DAEIIQLVNAK--HIPAYKLETLMETHERGVSIRRQLLSTKLAEPs-sLQYLPYRDYNYS 57
Rattus norvegicus            DAEIIQLVNAK--HIPAYKLETLMETHERGVSIRRQLLSAKLAEPs-sLQYLPYRDYNYS 57
Ustilago maydis              DEEIITLSQNG--KIAAYALEKVLQDHERAVRVRRALVSRASATQtleTSLLPHRDYDYG 58
Schizosaccharomyces pombe    DEEVVQLTLAK—-KIPLYALERVLKDVTRAVVIRRTVVSRSSRTKtleSSNCPVYHYDYS 58
Yarrowia lipolytica          DHEVVKLSLEG—-KLPLYALEKQLGDNTRAVGIRRSIISQQSNTKtleTSKLPYLHYDYD 58
Candida kefyr                NREVVSLVVAN—-KLPLYALEKQLGDTTRAVVVRRKALAILADAPvlaTERLPYKNYDYN 58
Kluyveromyces lactis         NKEVVSLVVNN—-KLPLYALEKQLGDTTRAVVVRRKALAILADAPvlsTERLPYKHYDYD 58
Candida glabrata             NKEVVSLVIHG—KLPLYALEKQLGDTTRAVCVRRKAISILADAPvlaTDRLPYKNYDYD  58
Saccharomyces HMG1           NKEVAALVIHG—-KLPLYALEKKLGDTTRAVAVRRKALSILAEAPvlaSDRLPYKNYDYD 58
Saccharomyces HMG2           NTEVSNLVVNG—-KLPLYSLEKKLEDTTRAVLVRRKALSTLAESPilvSEKLPFRNYDYD 58
Meyerozyma guilliermondii    DNEVTNLVVSG—-KLPLYALEKQLQDNTRAVVVRRKAIAQMAGVPsldSDRLPYMHYDYN 58
Clavispora lusitaniae        NSEVSSLVVAG—-QLPLYALEKQLGNNARAVVVRRKAIAKLANAPvleSNKLPHAHYDYD 58
Candida auris                NDEVSSLVVAG—-KLPLYALEKQLGDNLRAVVVRRKAIAKLANAPvldTDKLPFAHYDYD 58
Candida haemuloni            NDEVSSLVVAG—-KLPLYALEKQLQDNLRAVVVRRKAIAKLANAPvldTERLPYAHYDYD 58
Debaryomyces hansenii        NNEISSLVVAG—-KLPLYALEKQLADNTRAVLVRRKAIAKLANAPvldTKRLPYAHYDYD 58
Candida parapsilosis         NEEVTSLVVGG—-KLPLYALEKQLASNKRAVVVRRKAIAKLANAPvleTNRLPYAHYDYD 58
Candida orthopsilosis        NEEVTSLVVEG—-KLPLYALEKQLASNKRAVVVRRKAIAKLANAPvleTNRLPYAHYDYD 58
Candida tropicalis           NDEVSSLVVGG—-KLPLYALEKQLGDNKRAVAVRRKAIAKLANAPvleSNRLPYSHYDYD 58
Candida albicans             NDEVSSLVVGG—-KLPLYALEKQLADNKRAVIVRRKAIAKLANAPvldTNRLPYAHYDYD 58
Candida dubliniensis         NDEVSSLVVGG—-KLPLYALEKQLADNKRAVIVRRKAIAKLANAPvldTNRLPYAHYDYD 58

Pseudomonas mevalonii        DIANGMIENVIGTFELPYAVASNFQINGRDVLVPLVVEEPSIVAAASYMAKLARANGGFT 104
Arabidopsis thaliana         SILGQCCEMPVGYIQIPVGIAGPLLLDGYEYSVPMATTEGCLVASTNRGCKAMFISGGAT 115
Drosophila melanogaster      KVLNACCENVLGYVPIPVGYAGPLLLDGETYYVPMATTEGALVASTNRGCKALSVRG-VR 119
Homo sapiens                 LVMGACCENVIGYMPIPVGVAGPLCLDEKEFQVPMATTEGCLVASTNRGCRAIGLGGGAS 117
Mus musculus                 LVMGACCENVIGYMPIPVGVAGPLCLDGKEYQVPMATTEGCLVASTNRGCRAISLGGGAS 117
```

```
Rattus norvegicus           LVMGACCENVIGYMPIPVGVAGPLCLDGKEYQVPMATTEGCLVASTNRGCRAISLGGGAS 117
Ustilago maydis             KVMGACCENVVGYMPIPLGIAGPLNIDGQFMPIPMATTEGTLVASTSRGCKALNAGGGVT 118
Schizosaccharomyces pombe   RVLNACCENVIGYMPLPLGVAGPLIIDGKPFYIPMATTEGALVASTMRGCKAINAGGGAV 118
Yarrowia lipolytica         RVFGACCENVIGYMPLPVGVAGPMNIDGKNYHIPMATTEGCLVASTMRGCKAINAGGGVT 118
Candida kefyr               RVFGACCENVIGYMPLPVGVIGPLMIDGVYYHIPMATTEGCLVASAMRGCKAINSGGGVT 118
Kluyveromyces lactis        RVFGACCENVIGYMPLPVGVIGPLMIDGVYYHIPMATTEGCLVASAMRGCKAMNAGGGVQ 118
Candida glabrata            RVFGACCENVIGYMPLPVGVIGPLVIDGVSYHIPMATTEGCLVASAMRGCKAINAGGGVT 118
Saccharomyces HMG1          RVFGACCENVIGYMPLPVGVIGPLVIDGTSYHIPMATTEGCLVASAMRGCKAINAGGGAT 118
Saccharomyces HMG2          RVFGACCENVIGYMPIPVGVIGPLIIDGTSYHIPMATTEGCLVASAMRGCKAINAGGGAT 118
Meyerozyma guilliermondii   RVFGACCENVIGYMPIPVGVAGPLNIDGKSYHIPMATTEGCLVASTMRGCKAINAGGGVS 118
Clavispora lusitaniae       RVFGACCENVIGYMPLPVGVAGPLIIDGTPYHIPMATTEGCLVASTMRGCKAINAGGGVE 118
Candida auris               RVFGACCENVIGYMPLPVGVAGPLIIDGVPYHIPMATTEGCLVASTMRGCKAINSGGGVQ 118
Candida haemuloni           RVFGACCENVIGYMPLPVGVAGPLIIDGTPYHVPMATTEGCLVASTMRGCKAINSGGGVQ 118
Debaryomyces hansenii       RVFGACCENVIGYMPLPVGVAGPLIIDGKPYHIPMATTEGCLVASTMRGCKAINAGGGVE 118
Candida parapsilosis        RVFGACCENVIGYMPIPVGVAGPLIIDGKPYHIPMATTEGCLVASTMRGCKAINAGGGVE 118
Candida orthopsilosis       RVFGACCENVIGYMPIPVGVAGPLIIDGKPYHIPMATTEGCLVASTMRGCKAINAGGGVE 118
Candida tropicalis          RVFGACCENVIGYMPIPVGVAGPLVIDGKPYHIPMATTEGCLVASTMRGCKAINAGGGVE 118
Candida albicans            RVFGACCENVIGYMPLPVGVAGPLIIDEKPYHIPMATTEGCLVASTMRGCKAINAGGGVE 118
Candida dubliniensis        RVFGACCENVIGYMPLPVGVAGPLIIDEKPYHIPMATTEGCLVASTMRGCKAINAGGGVE 118

Pseudomonas mevalonii       TSSSAPLMHAQVQIVGIQDPLNARLSLLRRKDEI---IELANRKDQLLNSLGGGCRDIEV 161
Arabidopsis thaliana        STVLKDGMTRA--------P-VVRFASARRASELKFFLENPENFDTLAVVFNRSSRFARL 166
Drosophila melanogaster     SVVEDVGMTRA--------P-CVRFPSVARAAEAKSWIENDENYRVVKTEFDSTSRFGRL 170
Homo sapiens                SRVLADGMTRG--------P-VVRLPRACDSAEVKAWLETSEGFAVIKEAFDSTSRFARL 168
Mus musculus                SRVLADGMTRG--------P-VVRLPRACDSAEVKTWLETPEGFAVIKEAFDSTSRFARL 168
Rattus norvegicus           SRVLADGMSRG--------P-VVRLPRACDSAEVKSWLETPEGFAVVKEAFDSTSRFARL 168
Ustilago maydis             TVLTQDAMTRG--------P-ALEFPSVVQAAKAKRWIDSQEGAQTIKAAFDSTSRFARL 169
Schizosaccharomyces pombe   TVLTRDQMSRG--------P-CVAFPNLTRAGRAKIWLDSPEGQEVMKKAFNSTSRFARL 169
Yarrowia lipolytica         TVLTQDGMTRG--------P-CVSFPSLKRAGAAKIWLDSEEGLKSMRKAFNSTSRFARL 169
Candida kefyr               TVLTKDGMTRG--------P-CVRFPSLKRAGACKIWLDSEEGQNKIKKAFNSTSRFARL 169
Kluyveromyces lactis        TVLTKDGMTRG--------P-CVRFPSLARAGACKIWLDSEEGQNRVKKAFNSTSRFARL 169
Candida glabrata            TVLTKDGMTRG--------P-CVRFPSLTRAGACKIWLDSEEGQNQIKKAFDSTSRFARL 169
Saccharomyces HMG1          TVLTKDGMTRG--------P-VVRFPTLKRSGACKIWLDSEEGQNAIKKAFNSTSRFARL 169
Saccharomyces HMG2          TVLTKDGMTRG--------P-VVRFPTLIRSGACKIWLDSEEGQNSIKKAFNSTSRFARL 169
Meyerozyma guilliermondii   TVLTQDGMTRG--------P-CVSFPTLARAGAAKLWLDSEEGQKTIKKAFNSTSRFARL 169
Clavispora lusitaniae       TIITQDGMTRG--------P-CVSFSSLARAGACKLWLDSEEGQKTIKKAFNSTSRFARL 169
Candida auris               TILTQDGMTRG--------P-CVSFPSLARAGACKLWLDSEEGQRTIKKAFNSTSRFARL 169
Candida haemuloni           TILTQDGMTRG--------P-CVSFPSLARAGACKLWLDSEDGQRTIKKAFNSTSRFARL 169
Debaryomyces hansenii       SVLTQDGMTRG--------P-CVSFPSLSRAGACKLWLDSEEGQKTIKKAFNSTSRFARL 169
Candida parapsilosis        TVLTKDGMTRG--------P-CVRFPTLSRAGAAKLWIDSEEGQKTIKKAFNSTSRFARL 169
Candida orthopsilosis       TVLTKDGMTRG--------P-CVKFPTLARAGAAKLWIDSEEGQKTIKKAFNSTSRFARL 169
Candida tropicalis          TVLTKDGMTRG--------P-CVRFPTLKRAGAAKLWIDSEEGQTTIKKAFNSTSRFARL 169
Candida albicans            TVLTRDGMTRG--------P-CVRFPTLKRAGAAKLWIDSEQGQATIKKAFNSTSRFARL 169
Candida dubliniensis        TVLTRDGMTRG--------P-CVRFPTLKRAGAAKLWIDSEQGQATIKKAFNSTSRFARL 169

Pseudomonas mevalonii       HTFADTPRGPMLVAHLIVDVRDAMGANTVNTMAEAVAPLMEAITGGQVRLRILSNLADLR 221
Arabidopsis thaliana        QSVKCTIAGKNAYVRFCCSTGDAMGMNMVSKGVQNVLEYLTDD---FPDMDVIGISGNFC 223
Drosophila melanogaster     KDCHIAMDGPQLYIRFVAITGDAMGMNMVSKGAEMALRRIQL--Q-FPDMQIISLSGNFC 227
Homo sapiens                QKLHTSIAGRNLYIRFQSRSGDAMGMNMISKGTEKALSKLHEY---FPEMQILAVSGNYC 225
Mus musculus                QKLHVTMAGRNLYIRFQSRTGDAMGMNMISKGTEKALLKLQEF---FPDMQILAVSGNYC 225
Rattus norvegicus           QKLHVTLAGRNLYIRLQSKTGDAMGMNMISKGTEKALLKLQEF---FPELQILAVSGNYC 225
Ustilago maydis             SSLRCVLAGRTLYVRFATSTGDAMGMNMISKGVEKALGLMTEQY--FpEMKVLSLSGNYC 227
Schizosaccharomyces pombe   QHIKTALAGTRLFIRFCTSTGDAMGMNMISKGVEHALVVMSNDAG-fdDMQVISVSGNYC 228
Yarrowia lipolytica         QSLHSTLAGNLLFIRFRTTTGDAMGMNMISKGVEHSLAVMVKEYG-fpDMDIVSVSGNYC 228
Candida kefyr               QHVQTALAGDLLFIRFRTTTGDAMGMNMISKGVEFSLHQMVEEYG-wkDMEIVSVSGNYC 228
Kluyveromyces lactis        QHIQTALAGDLLFIRFRTTTGDAMGMNMISKGVEFSLKQMVEEFG-wnDMEIVSVSGNFC 228
Candida glabrata            QHAQTALAGDLLFIRFRTTTGDAMGMNMISKGVEFVLKQMVEEFG-whDMEIVSVSGNYC 228
Saccharomyces HMG1          QHIQTCLAGDLLFMRFRTTTGDAMGMNMISKGVEYSLKQMVEEYG-weDMEVVSVSGNYC 228
Saccharomyces HMG2          QHIQTCLAGDLLFMRFRTTTGDAMGMNMISKGVEYSLKQMVEEYG-weDMEVVSVSGNYC 228
Meyerozyma guilliermondii   QHIKTALAGTLLFIRFRTTTGDAMGMNMISKGVEYCLKHMVEECG-fkDMTVISVSGNYC 228
Clavispora lusitaniae       QHIKTAIAGTLLFIRFKTTTGDAMGMNMISKGVEYSLKYMAEECG-wdDMSVIAVSGNYC 228
Candida auris               QHVKTAIAGTLLFIRFKTTTGDAMGMNMISKGVEHSLKFMMEECG-feDMSVIAVSGNYC 228
Candida haemuloni           QHVKTAIAGTLLFIRFKTTTGDAMGMNMISKGVEHSLKFMSEECG-fdDMSVIAVSGNYC 228
Debaryomyces hansenii       QHIQTALAGTLLFIRFRTTTGDAMGMNMISKGVEYSLKYMSEECD-wsDMEVISVSGNYC 228
Candida parapsilosis        QHIQTSLAGCLLFIRFRTTTGDAMGMNMISKGVEYSLKYMVEECG-yeDMEIISVSGNYC 228
Candida orthopsilosis       QHIQTALAGCLLFIRFRTTTGDAMGMNMISKGVEHSLKYMVEECG-yeDMEIISVSGNYC 228
Candida tropicalis          QHIQTALAGTSLFVRFRTTTGDAMGMNMISKGVEYSLKYMVEECG-wdDMEVVSVSGNYC 228
Candida albicans            QHIQTALAGTSLFIRFRTTTGDAMGMNMISKGVEYSLKYMVEECG-wdDMEIVSVSGNYC 228
```

```
Candida dubliniensis        QHIQTALAGTSLFIRFRTTTGDAMGMNMISKGVEYSLKYMVEECG-wdDMEIVSVSGNYC 228

Pseudomonas mevalonii       LARAQVRITPQQLETAEFSGE-----AVIEG-----ILDAYAFAAVDPYR----A----- 262
Arabidopsis thaliana        SD-----KKPAAVNWIEGRGKSVVCEAVIRGEIVNKVLKTSVAALVELNMLKNLAGSAVA 278
Drosophila melanogaster     CD-----KKPAAINWIKGRGKRVVTECTISAATLRSVLKTDAKTLVECNKLKNMGGSAMA 282
Homo sapiens                TD-----KKPAAINWIEGRGKSVVCEAVIPAKVVREVLKTTTEAMIEVNINKNLVGSAMA 280
Mus musculus                TD-----KKPAAINWIEGRGKTVVCEAVIPAKVVREVLKTTTEAMVDVNINKNLVGSAMA 280
Rattus norvegicus           TD-----KKPAAINWIEGRGKTVVCEAVIPAKVVREVLKTTTEAMVDVNINKNLVGSAMA 280
Ustilago maydis             TD-----KKPAAINWIEGRGKSVVAEAVVPGNVVRSVLKCTVRDLVNLNTKKNLIGSAMA 282
Schizosaccharomyces pombe   TD-----KKPAAINWIDGRGKSVIAEAIIPGDAVKSVLKTTVEDLVKLNVDKNLIGSAMA 283
Yarrowia lipolytica         TD-----KKPAAINWIEGRGKSVVAEATIPAHIVKSVLKSEVDALVELNISKNLIGSAMA 283
Candida kefyr               MD-----KKPAAINWIEGRGKSVVAEANIPGDVVRKVLKSDVKALVDLNISKNLIGSAMA 283
Kluyveromyces lactis        MD-----KKPAAINWIEGRGKSVVAEATIPGDVVRKVLKSDVKALVDLNISKNLIGSAMA 283
Candida glabrata            TD-----KKPAAINWIEGRGKSIVAEAIIPGDVVRKVLKSDVSALVELNISKNLIGSAMA 283
Saccharomyces HMG1          TD-----KKPAAINWIEGRGKSVVAEATIPGDVVRKVLKSDVSALVELNIAKNLVGSAMA 283
Saccharomyces HMG2          TD-----KKPAAINWIEGRGKSVVAEATIPGDVVKSVLKSDVSALVELNISKNLVGSAMA 283
Meyerozyma guilliermondii   TD-----KKPSAINWIEGRGKSVVAEARIPADVVKNVLKSDVDALVELNVSKNLVGSAMA 283
Clavispora lusitaniae       TD-----KKPAAINWIEGRGKSVVAEARIPSEVVQKVLKSDVDALVELNISKNLIGSAMA 283
Candida auris               TD-----KKPAAINWIEGRGKSVVAEARIPADVVRKVLKSDVDALVELNVSKNLVGSAMA 283
Candida haemuloni           TD-----KKPAAINWIEGRGKSVVAEARIPKDVVEKVLKSDVDALVELNVSKNLVGSAMA 283
Debaryomyces hansenii       TD-----KKPAAINWIEGRGKSVVAEARIPASVVQKVLKSDVDALVELNISKNLVGSAMA 283
Candida parapsilosis        SD-----KKPAAINWLEGRGKSIVAEATIPADVVAKVLKSDVDALVELNISKNLVGSAMA 283
Candida orthopsilosis       SD-----KKPAAINWLEGRGKSIVAEATIPADVVAKVLKSDVDALVELNISKNLVGSAMA 283
Candida tropicalis          TD-----KKPAAINWIEGRGKSIVAAATIPAEVVTKVLKSDVDALVELNISKNLVGSAMA 283
Candida albicans            TD-----KKPAAINWIEGRGKSIVAAARIPADVVTKVLKSDVDALVELNISKNLVGSAMA 283
Candida dubliniensis        TD-----KKPAAINWIEGRGKSIVAAARIPADVVTKVLKSDVDALVELNISKNLVGSAMA 283

Pseudomonas mevalonii       ---ATHNKGIMNGIDPLIVATGNDWRAVEAGAHAYACRSGHYGSLTTWEKDNNGHLVGTL 319
Arabidopsis thaliana        GSLGGFNAHASNIVSAVFIATGQDPAQ--------NVESSQCITMMEAIND-GKDIHISV 329
Drosophila melanogaster     GSIGGNNAHAANMVTAVFLATGQDPAQ--------NVTSSNCSTAMECWAeNSEDLYMTC 334
Homo sapiens                GSIGGYNAHAANIVTAIYIACGQDAAQ--------NVGSSNCITLMEASGpTNEDLYISC 332
Mus musculus                GSIGGYNAHAANIVTAIYIACGQDAAQ--------NVGSSNCITLMEASGpTNEDLYISC 332
Rattus norvegicus           GSIGGYNAHAANIVTAIYIACGQDAAQ--------NVGSSNCITLMEASGpTNEDLYISC 332
Ustilago maydis             GSVGGFNAHAANILTAIYLATGQDPAQ--------NVESSNCITLMEAIND-DEDLLITV 333
Schizosaccharomyces pombe   GSVGGFNAHAANIVTAVYLATGQDPAQ--------NVESSNCITLMDNV---DGNLQLSV 332
Yarrowia lipolytica         GSVGGFNAHAANLVTAIYLATGQDPAQ--------NVESSNCITLMSNV---DGNLLISV 332
Candida kefyr               GSIGGFNAHASNLVTAVYLALGQDPAQ--------NVESSNCMTLMKEV---DGDLRISV 332
Kluyveromyces lactis        GSVGGFNAHASNLVSAVFLALGQDPAQ--------NVESSNCITLMKEV---DGDLRISV 332
Candida glabrata            GSVGGFNAHAANLVTAVYLALGQDPAQ--------NVESSNCMTLMNDV---NGDLKISV 332
Saccharomyces HMG1          GSVGGFNAHAANLVTAVFLALGQDPAQ--------NVESSNCITLMKEV---DGDLRISV 332
Saccharomyces HMG2          GSVGGFNAHAANLVTALFLALGQDPAQ--------NVESSNCITLMKEV---DGDLRISV 332
Meyerozyma guilliermondii   GSVGGFNAQAANLVTAVFLACGQDPAQ--------NVESSNCITLVNKV---DGDLVISV 332
Clavispora lusitaniae       GSVGGFNAHAANLVTAVYLACGQDPAQ--------NVESSNCMTLMNRL-P-NGDLQISV 333
Candida auris               GSVGGFNAHAANLVTAVYLACGQDPAQ--------NVESSNCITLMNKV---GDDLQISV 332
Candida haemuloni           GSVGGFNAHAANLVTAVFLACGQDPAQ--------NVESSNCITLMNKV---GDDLQISV 332
Debaryomyces hansenii       GSVGGFNAHAANLVTAVFLACGQDPAQ--------NVESSNCITLMKNV---GGDLQISV 332
Candida parapsilosis        GSVGGFNAHASNLVTAVYLACGQDPAQ--------NVESSNCITLMNKHKV-TGDLQISV 334
Candida orthopsilosis       GSVGGFNAHASNLVTAVYLACGQDPAQ--------NVESSNCITLMNKHKV-TGDLQISV 334
Candida tropicalis          GSVGGFNAHAANLVTAVYLACGQDPAQ--------NVESSNCITLMEKNKE-TGDLQISV 334
Candida albicans            GSVGGFNAHAANLVTAVYLACGQDPAQ--------NVESSNCITLMEKDKE-TGDLNVSV 334
Candida dubliniensis        GSVGGFNAHAANLVTAVYLACGQDPAQ--------NVESSNCITLMEKDKE-TGDLNVSV 334

Pseudomonas mevalonii       EMP-MPVGLVGGATKTHPLAQLSLRILGVKTA---------QALAEIAVAVGLAQNLGAM 369
Arabidopsis thaliana        TMPSIEVGTVGGGTQLASQS-ACLNLLGVKGASTESPGMNARRLATIVAGAVLAGELSLM 388
Drosophila melanogaster     TMPSLEVGTVGGGTGLPGQS-ACLEMLGVRGAHATRPGDNAKKLAQIVCATVMAGELSLM 393
Homo sapiens                TMPSIEIGTVGGGTNLLPQQ-ACLQMLGVQGACKDNPGENARQLARIVCGTVMAGELSLM 391
Mus musculus                TMPSIEIGTVGGGTNLLPQQ-ACLQMLGVQGACKDNPGENARQLARIVCGTVMAGELSLM 391
Rattus norvegicus           TMPSIEIGTVGGGTNLLPQQ-ACLQMLGVQGACKDNPGENARQLARIVCGTVMAGELSLM 391
Ustilago maydis             SMPSIEVGTVGGGTVLPPQR-SMLEMMGIAGAHSTTPGANAQRLARIIAASVMAGELSLM 392
Schizosaccharomyces pombe   SMPSIEVGTIGGGTVLEPQG-AMLDLLGVRGAHMTSPGDNSRQLARVVAAAVMAGELSLC 391
Yarrowia lipolytica         SMPSIEVGTIGGGTILEPQG-AMLEMLGVRGPHIETPGANAQQLARIIASGVLAAELSLC 391
Candida kefyr               SMPSIEVGTIGGGTILEPQS-AMLDLLGVRGPHPTEPGKNARQLAKIVASAVMAGELSLC 391
Kluyveromyces lactis        SMPSIEVGTIGGGTILEPQG-AMLDLLGVRGPHPTQPGNNARQLAKIVASAVMAGELSLC 391
Candida glabrata            SMPSIEVGTIGGGTVLDPQG-AMLDLLGVRGPHPTNPGANARQLAKIVACAVLAGELSLC 391
Saccharomyces HMG1          SMPSIEVGTIGGGTVLEPQG-AMLDLLGVRGPHATAPGTNARQLARIVACAVLAGELSLC 391
Saccharomyces HMG2          SMPSIEVGTIGGGTVLEPQG-AMLDLLGVRGPHPTEPGANARQLARIIACAVLAGELSLC 391
Meyerozyma guilliermondii   SMPSIEVGTIGGGTILEPQG-AMLDLLRVKGPHPTNPGENARQLARVVASAVLAAELSLC 391
Clavispora lusitaniae       SMPSIEVGTIGGGTILEPQG-AMLELLGVRGPHPTNPGDNSRRLACIVASAVLAAELSLC 392
```

```
Candida auris              SMPSIEVGTIGGGTILEAQG-SMLDLLGVRGPHPKNPGDNSRRLACIVASAVLAAELSLC 391
Candida haemuloni          SMPSIEVGTIGGGTILEAQG-SMLDLLGVRGPHPNNPGDNSRRLACIVASTVLAAELSLC 391
Debaryomyces hansenii      SMPCIEVGTIGGGTILEPQG-AMLDLLGVRGPHPTNPGDNARQLARIVASAVLAAELSLC 391
Candida parapsilosis       SMPSIEVGTIGGGTILEPQG-AMLDLLGVRGPHPTNPGDNARQLAKIVASAVLAAELSLC 393
Candida orthopsilosis      SMPSIEVGTIGGGTILEPQG-AMLDLLGVRGPHPTNPGDNARQLAKIVASAVLAAELSLC 393
Candida tropicalis         SMPSIEVGTIGGGTILEPQG-AMLELLGVRGPHPTNPGDNARQLAKIVASAVLAAELSLC 393
Candida albicans           SMPSIEVGTIGGGTILEPQG-AMLDLLGVRGPHPTNPGANAQQLAKIVASAVLAAELSLC 393
Candida dubliniensis       SMPSIEVGTIGGGTILEPQG-AMLDLLGVRGPHPTNPGENARQLAKIVASAVLAAELSLC 393

Pseudomonas mevalonii      RALATEGIQRGHMALHARNIAVVAGARGDEVDWVARQLVEYHDVRADRAVALLKQKRGQ 428
Arabidopsis thaliana       SAIAAGQLVRSHMKYNRS----------------------------------------- 406
Drosophila melanogaster    AALVNSDLVKSHMRHNR------------------------------------------ 410
Homo sapiens               AALAAGHLVKSHMIHNRS----------------------------------------- 409
Mus musculus               AALAAGHLVRSHMVHNRS----------------------------------------- 409
Rattus norvegicus          AALAAGHLVRSHMVHNRS----------------------------------------- 409
Ustilago maydis            GALCAGHLIQAHMKHNRS----------------------------------------- 410
Schizosaccharomyces pombe  SALASGHLVKSHIGLNRS----------------------------------------- 409
Yarrowia lipolytica        SALAAGHLVQSHMTHNR------------------------------------------ 408
Candida kefyr              SALAAGHLVQSHMVHNR------------------------------------------ 408
Kluyveromyces lactis       SALAAGHLVQSHMVHNR------------------------------------------ 408
Candida glabrata           AALAAGHLVQSHMTHNR------------------------------------------ 408
Saccharomyces HMG1         AALAAGHLVQSHMTHNR------------------------------------------ 408
Saccharomyces HMG2         SALAAGHLVQSHMTHNR------------------------------------------ 408
Meyerozyma guilliermondii  SALAAGHLVQSHMQHNR------------------------------------------ 408
Clavispora lusitaniae      SALAAGHLVQSHMQHNR------------------------------------------ 409
Candida auris              SALAAGHLVQSHMQHNR------------------------------------------ 408
Candida haemuloni          SALAAGHLVQSHMQHNR------------------------------------------ 408
Debaryomyces hansenii      SALAAGHLVQSHMQHNR------------------------------------------ 408
Candida parapsilosis       SALAAGHLVQSHMQHNR------------------------------------------ 410
Candida orthopsilosis      SALAAGHLVQSHMQHNR------------------------------------------ 410
Candida tropicalis         SALAAGHLVQSHMQHNR------------------------------------------ 410
Candida albicans           SALAAGHLVQSHMQHNRS----------------------------------------- 411
Candida dubliniensis       SALAAGHLVQSHMQHNR------------------------------------------ 410
```

Supplementary Figure S1. Multiple sequence alignment of the catalytic portions of the HMGRs from different organisms. The dimerization domain is highlighted in yellow, the substrate binding domain in green, and the cofactor binding domain in cyan.

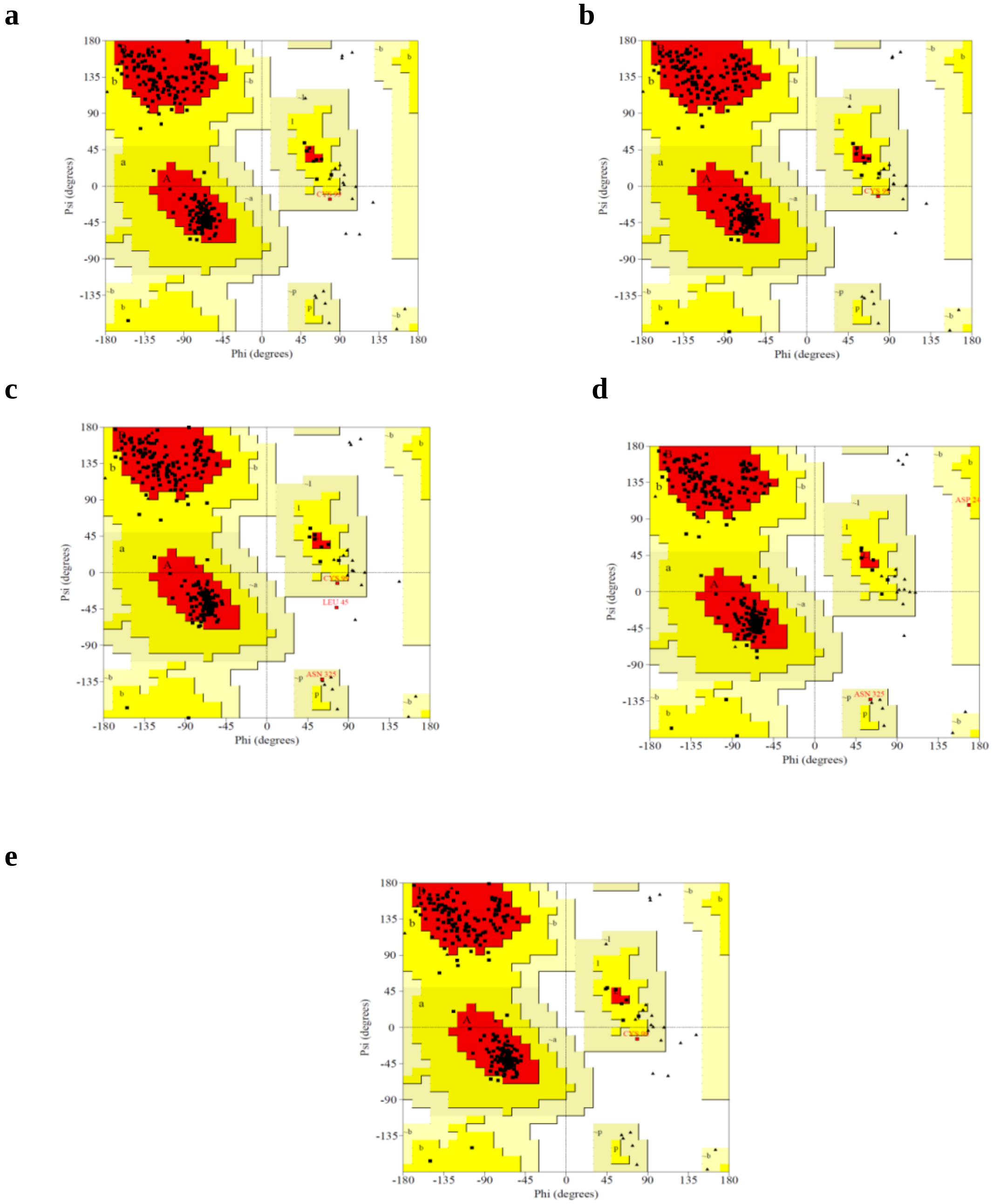

Supplementary Figure S2. Ramachandran plots of the mutated HMGR proteins of *Candida glabrata* (*Cg*HMGR): (**a**) *Cg*HMGRE680Q, (**b**) *Cg*HMGRE711Q, (**c**) *Cg*HMGRD805A, (**d**) CgHMGRM807R, and (**e**) *Cg*HMGRE680Q-M807R. The residues are portrayed with black squares, being A, B, and L in the favored region (red zones), a, b, l, and p in the allowed region (yellow zones), and ~ a, ~ b, ~ l, and ~ p in the generously allowed region (beige zones). No residues were found in the disallowed region (white zones).

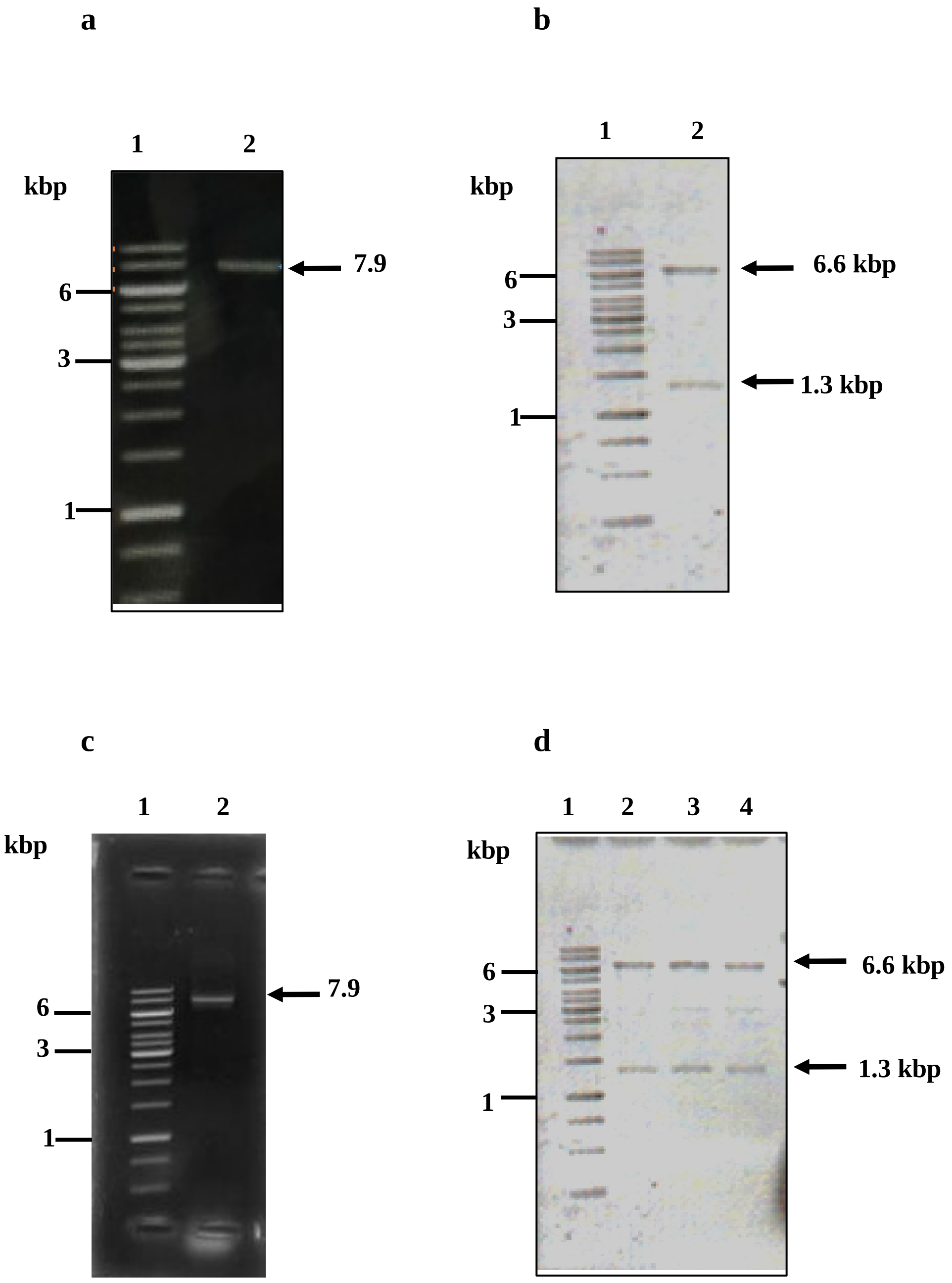

Supplementary Figure S3. Through amplification and digestion, the *Cg*HMGR mutants obtained by mutagenic PCR were verified. (**a**) Amplification of the *Cg*HMGR mutant: Lane 1, the 1 kbp molecular size marker; lane 2, the 7.9 kbp product. (**b**) Digestion of the plasmids of the HMGRCg mutants by the BamHI and Sall enzymes: Lane 1, the1 kbp marker; lane 2, the products of double digestion (6.6 and 1.3 kbp). (**c**) Full-length gel original-amplification PCR, corresponding to Fig. S2a. (**d**) Full-length gel original-digestion plasmid, corresponding to Fig. S2b.

a

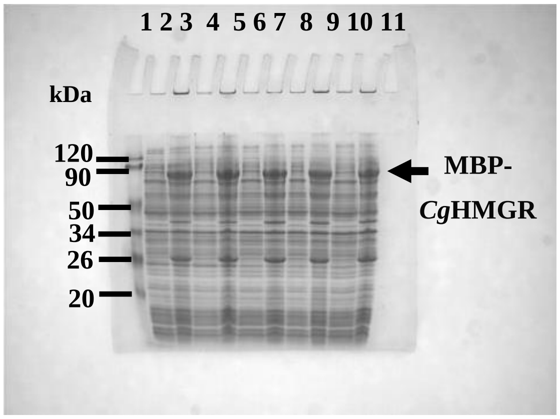

b

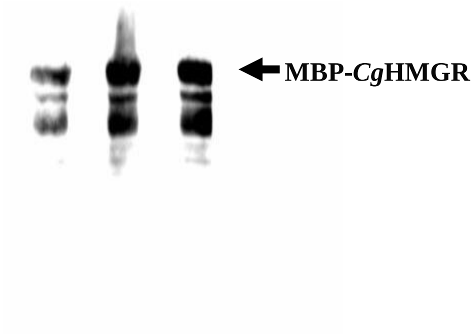

Supplementary Figure S4. The full-length gel/blots are displayed. (**a**) The full-length original gel, corresponding to Fig. 6b in the main text. (**b**) The full-length original blots, corresponding Fig.6c in the main text.

simvastatin | **alpha-asarone** | **HMG-CoA**

Wild-type

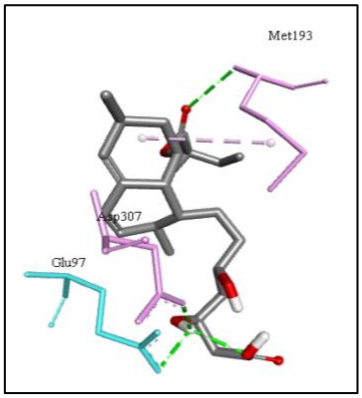

-10.71 Kcal/mol

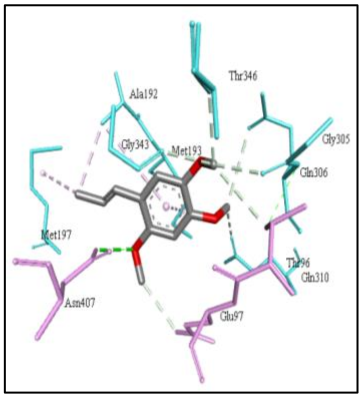

-6.4 Kcal/mol

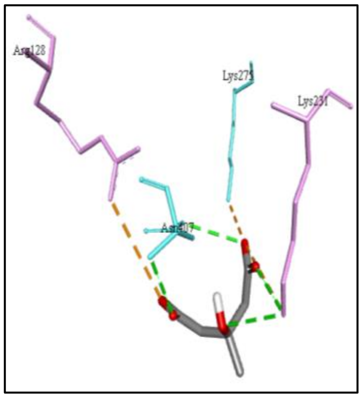

-6.48 Kcal/mol

***Cg*HMGR E680Q**

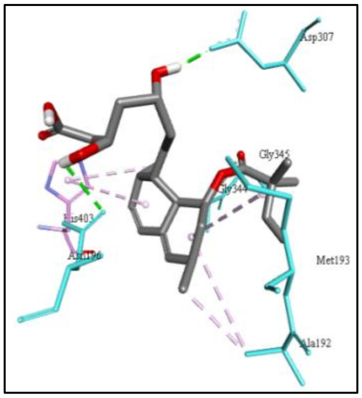

-10.18 Kcal/mol

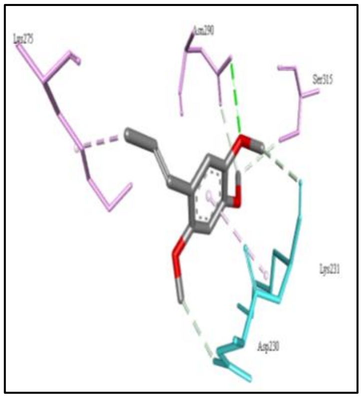

-5.81 Kcal/mol

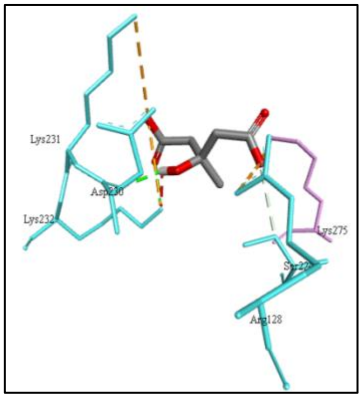

-6.31 Kcal/mol

***Cg*HMGR E711Q**

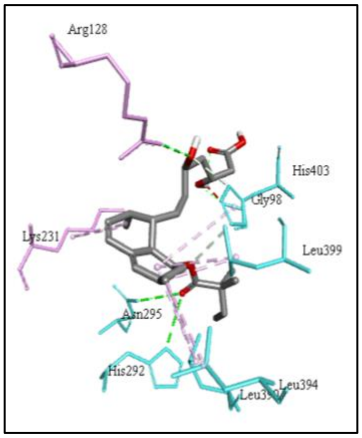

-7.14 Kcal/mol

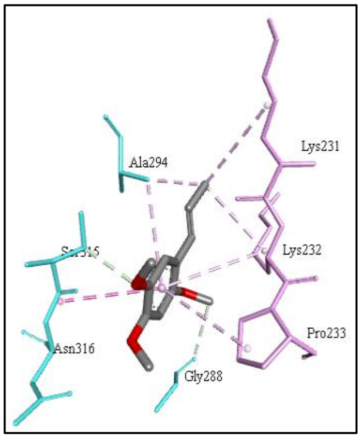

-5.7 Kcal/mol

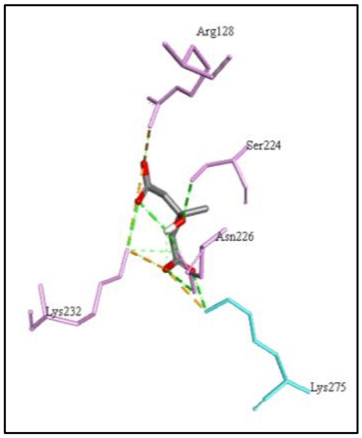

-6.05 Kcal/mol

***Cg*HMGR D805A**

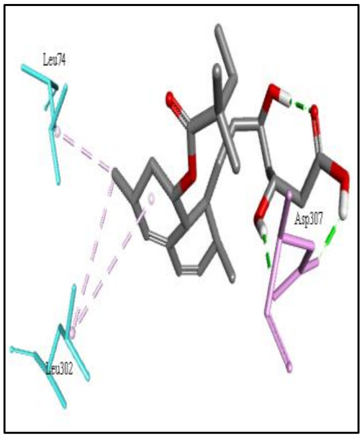

-9.54 Kcal/mol

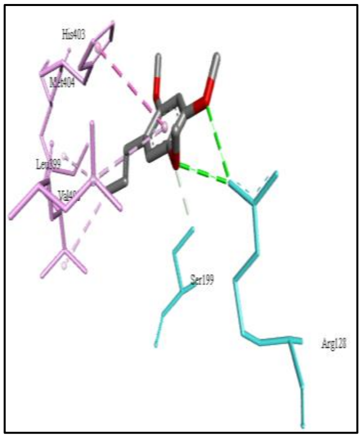

-6.33 Kcal/mol

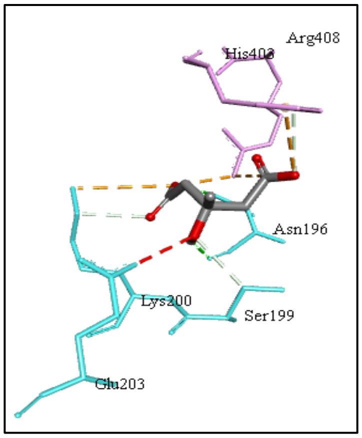

-6.26 Kcal/mol

***Cg*HMGR M807R**

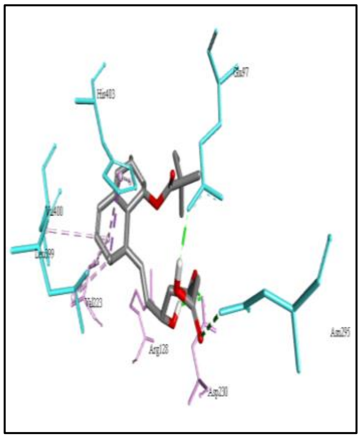

-9.78 Kcal/mol

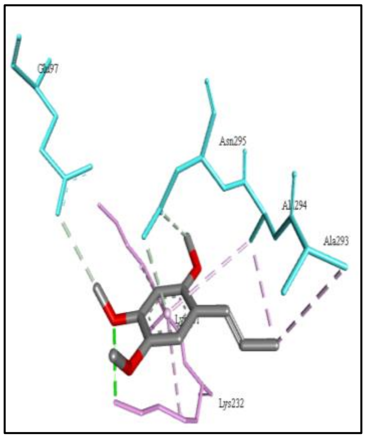

-6.86 Kcal/mol

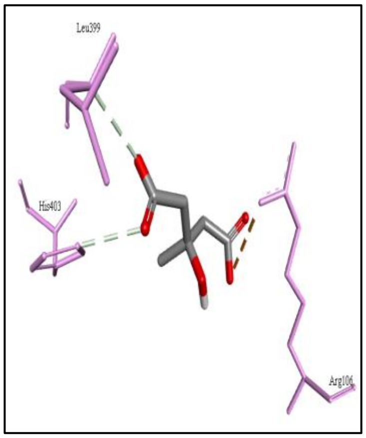

-6.35 Kcal/mol

***Cg*HMGR E680Q-M807R**

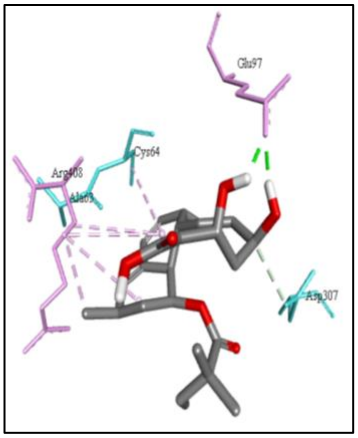

-7.36 Kcal/mol

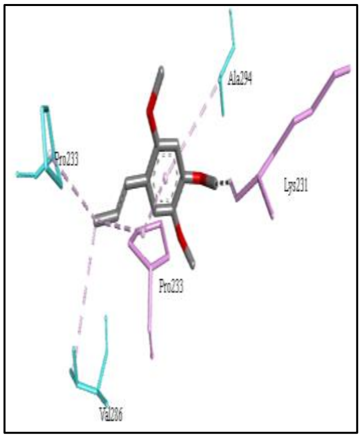

-5.8 Kcal/mol

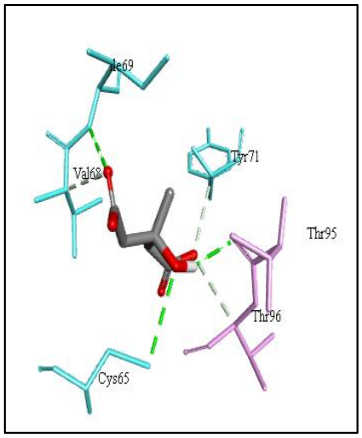

-6.13 Kcal/mol

Figure S5. 3D representation of the molecular interactions of simvastatin, alpha-asarone, and HMG-CoA with the active site of the wild-type and mutant *Cg*HMGR proteins. The amino acids of monomer α are depicted in pink and those of monomer β in cyan. The dotted lines denote the following bonds: conventional hydrogen (green), carbon-hydrogen (gray), pi-anion (orange), and pi-alkyl (pink). Interaction energies are expressed in Kcal/mol. Made using Discovery Studio software.

```
CgHMGR WT            ACTACGATTATGATAGAGTCTTTGGTGCTTGTTGTGAAAATGTGATTGGTTACATGCCAT
CgHMGRE680Q          ACTACGATTATGATAGAGTCTTTGGTGCTTGTTGTCAAAATGTGATTGGTTACATGCCAT

CgHMGR WT            CCACAACTGAAGGTTGCTTGGTTGCATCTGCCATGCGTGGTTGCAAGGCTATAAATGCCG
CgHMGRE711Q          CCACAACTCAAGGTTGCTTGGTTGCATCTGCCATGCGTGGTTGCAAGGCTATAAATGCCG

CgHMGR WT            CTGCTCTTGCTGGTGACTTACTATTTATTCGTTTTAGAACTACAACTGGTGATGCGATGG
CgHMGRD805A          CTGCTCTTGCTGGTGACTTACTATTTATTCGTTTTAGAACTACAACTGGTGCTGCGATGG

CgHMGR WT            TTTGTTTTAACACAAATTCAACACCTTTAGAAATCATATTCATACCCATCGCATCACCAG
CgHMGRM807R          TTTGTTTTAACACAAATTCAACACCTTTAGAAATCATATTCATACCCCTCGCATCACCAG
CgHMGRE680Q-M807R    TTTGTTTTAACACAAATTCAACACCTTTAGAAATCATATTCATACCCCTCGCATCACCAG

CgHMGR WT            TCACATTTTCACAACAAGCACCAAAGAATCTATCAAAATCGTATTCTTGTAAGGAAAACA
CgHMGRM807R          ------------------------------------------------------------
CgHMGRE680Q-M807R    TCACATTTTGACAACAAGCACCAAGAATCTATTCAAAATCGTAATTTTTGTTAGGGA---
```

Figure S6. Alignment of the nucleotide sequences, comparing wild-type *Candida glabrata* HMGR (*Cg*HMGR) to the mutants *Cg*HMGRE680Q, *Cg*HMGRE711Q, *Cg*HMGRD805A, *Cg*HMGRM807R, and *Cg*HMGRE680Q-M807R. The triplets coding for the amino acids that were the target of mutation are highlighted in yellow. The mutated nucleotide base (marked in fuchsia) changed from G to C for *Cg*HMGRE680Q and *Cg*HMGRE711Q, from A to C for *Cg*HMGRD805A and *Cg*HMGRM807R, and from A to C and C to G for *Cg*HMGRE680Q-M807R. The alignment was performed on the MUSCLE program.

Supplementary Table S1. Statistics for the Ramachandran plots constructed for the protein models of mutated HMGR of *Candida glabrata* (*Cg*HMGR).

**Ramachandran plot statistics**	***Cg*HMGRE680Q**		***Cg*HMGRE711Q**		***Cg*HMGRD805A**		***Cg*HMGRM807R**		***Cg*HMGRE7680Q-M807R**	
	Residues	%	Residues	%	Residues	%	Residues	%	Residues	%
Most favored regions	331	95.1	329	94.5	324	93.1	326	93.7	327	94
Allowed regions	16	4.6	18	5.2	21	6	20	5.7	20	5.7
Generously allowed regions	1	0.3	1	0.3	2	0.6	2	0.6	1	0.3
Disallowed regions	0	0	0	0	1	0.3	0	0	0	0
Non-glycine and non-proline residues	348	100	348	100	348	100	348	100	348	100
End residues (excluding Gly and Pro)	2	—	2	—	2	—	2	—	2	—
Glycine residues	41	—	41	—	41	—	41	—	41	—
Proline residues	17	—	17	—	17	—	17	—	17	—

*Cg*HMGRE680Q

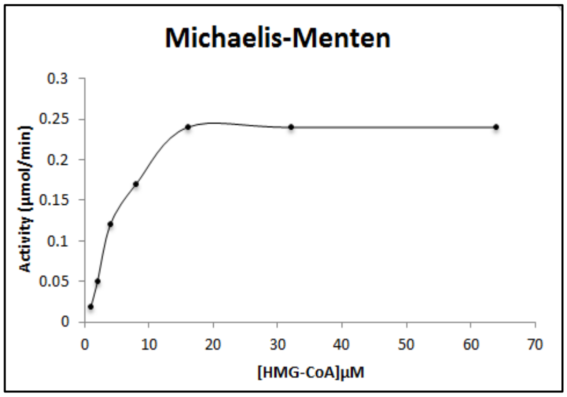

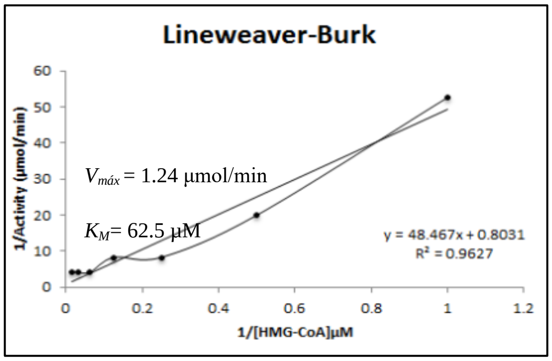

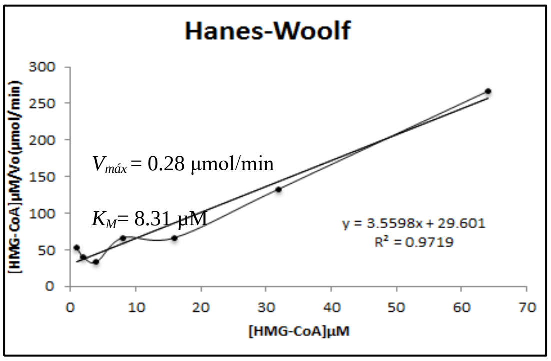

*Cg*HMGRE711Q

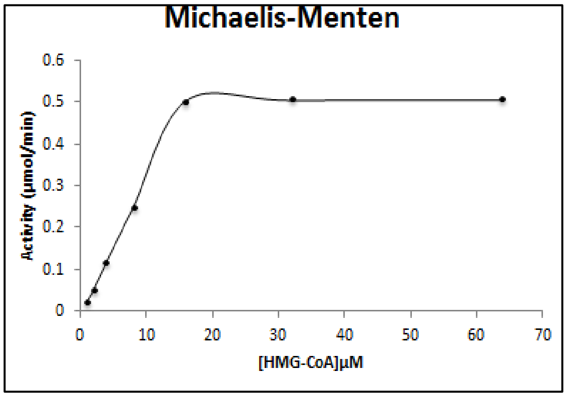

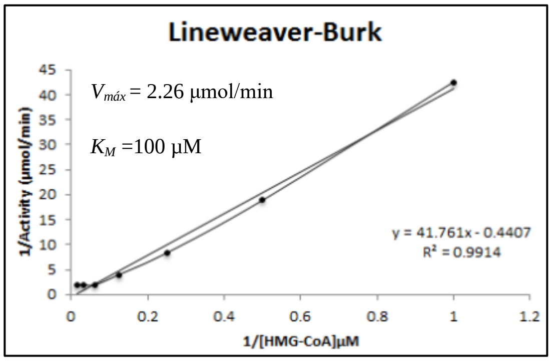

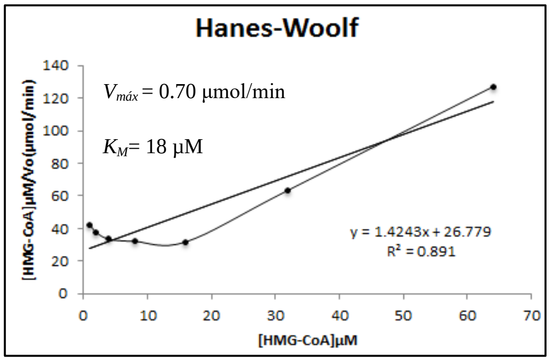

***Cg*HMGRD805A**

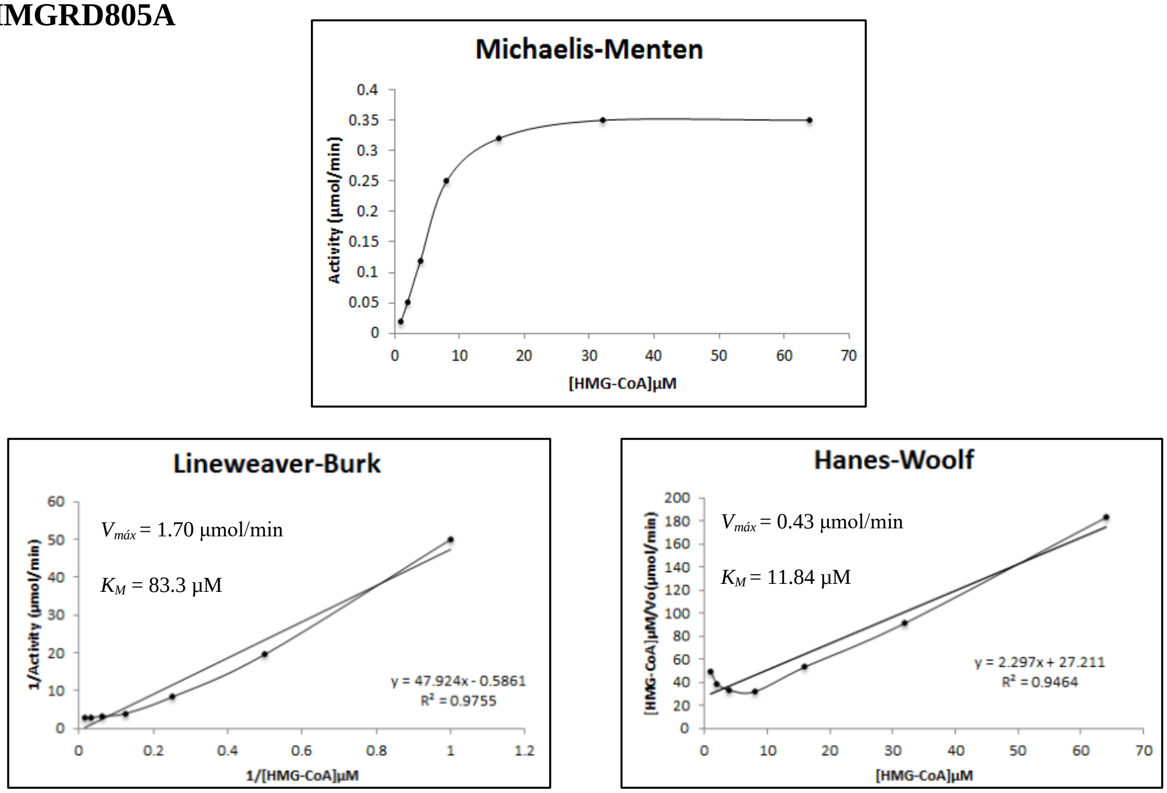

Figure S7. Michaelis-Menten, Lineweaver-Burk and Hanes-Woolf plots obtained with the MBP-*Cg*HMGR activity assay.
